# Fabrication of 1-octane sulphonic acid modified nanoporous graphene with tuned hydrophilicity for decontamination of industrial wastewater from organic and inorganic contaminants†

**DOI:** 10.1039/d3ra02602g

**Published:** 2023-07-20

**Authors:** Shahbaz Ali Mallah, Huma Shaikh, Najma Memon, Sehrish Qazi

**Affiliations:** a National Centre of Excellence in Analytical Chemistry, University of Sindh Jamshoro 76080 Pakistan huma.hashu@gmail.com

## Abstract

This research work is based on the fabrication of a graphene oxide-based composite (GOBC) to remove the maximum number of contaminants from different industrial effluents. The GO was first intercalated with 1-octanesulphonic acid sodium salt and subjected to microwave irradiation to produce GOBC. Fixed-bed column tests and Jar-tests were performed for removal of the most harmful endocrine disrupting compounds (EDCs) such as bisphenol A, bisphenol S, endosulphan, beta-estradiol, dyes (methylene blue and violate) and toxic metal ions such as Pb^2+^, Li^+^, Ni^2+^, Co^2+^, Cr^6+^, Zn^2+^, Cd^2+^, Hg^2+^, Cu^2+^, and As^5+^*via* adsorption. The prepared material was thoroughly characterized for its unique functional and structural properties. The results obtained from Fourier transform infrared spectroscopy, Brunauer–Emmett–Teller, scanning electron microscopy, Raman spectroscopy, water contact angle and X-ray diffraction analysis confirmed the successful preparation of GOBC using the proposed intercalation/microwave method. The water contact angle results showed decreased hydrophilicity of GOBC as compared to GO as the contact angle of GOBC (77.75°) was higher than that of GO (53.98°). The effects of main column parameters such as bed height, initial analyte concentration and solution flow rate were investigated. The results revealed that shorter breakthrough time, and high adsorption capacity were obtained at high flow rates of 1 mL min^−1^, while longer breakthrough time and lower adsorption capacity were obtained at lower flow rates of 0.5 mL min^−1^. The effect of bed depth on the breakthrough curve of analyte adsorption was a steep breakthrough curve; or a shorter breakthrough time occurring at lower bed height. The adsorption data obeyed the Yoon–Nelson and Thomas models very well. The adsorption capacity for BPA, BPS, endosulphan, beta-estradiol, methylene blue and violate was found to be 307, 305, 260, 290, 230 and 195 mg g^−1^, respectively. The adsorption capacity of GOBC for toxic metal ions such as Pb^2+^, Li^+^, Ni^2+^, Co^2+^, Cr^6+^, Zn^2+^, Cd^2+^, Hg^2+^, Cu^2+^, and As^5+^ was found to be 156, 136, 126, 124, 118, 114, 82, 82, 72 and 72 mg g^−1^, respectively with excellent kinetics. The adsorption data obtained using Jar-tests revealed that GOBC obeys a Langmuir isotherm and a pseudo second order kinetics model. The analysis of industrial wastewater samples showed good removal efficiency of GOBC.

## Abstract 2

The fabricated graphene oxide-based composite (GOBC) can act as a universal adsorbent for the removal of maximum number of contaminants from different industrial effluents.

## Graphical abstract

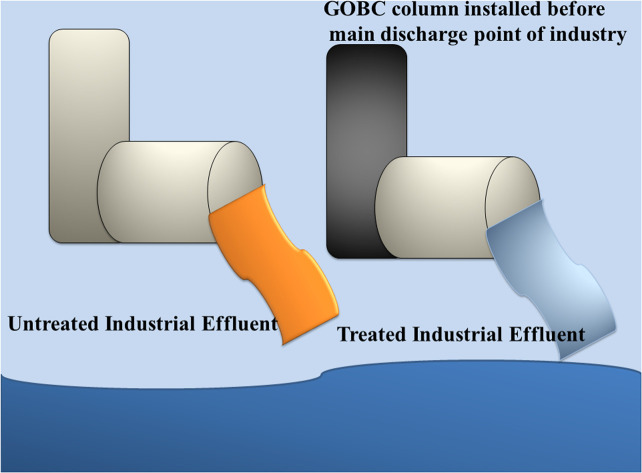

## 1. Introduction

The environment has paid a high price for urbanization and industrialization. Several synthetic/natural organic compounds are classified as highly toxic pollutants due to their ability to destroy aquatic and human life even at trace level concentrations. These chemicals can easily accumulate in the human body through breathing, eating and skin contact.

According to the United Nations (2006), two-third of the world's population will suffer from water shortages due to a continuous rise in water pollution by 2025.^1^ Industrial effluents and human activities such as unnecessary use of pesticide and fertilizers are the major sources of organic and inorganic contaminants in the aqueous environment. The industrial effluents contain large amounts of organic compounds such as pesticides, plasticizers, antibiotics, hydrocarbon, herbicides, dyes, phenols, proteins, and detergents that are directly discharged to the fresh water bodies. These industrial effluents also contain highly toxic metal ions. Among these toxins EDCs pose potential threat to aquatic and human life due to their extended half-life and ability to bioaccumulate in aquatic plants, animals and human body.^2^ Hence, there is need to maintain the clean water resources and limit the discharge of contaminated industrial effluent to fresh water bodies. Therefore, recent research work was focused on developing a universal adsorbent that can treat greater volumes of variety of industrial effluents.

Many wastewater treatment systems have been developed to overcome this challenging task. The continuous advancement in nanoscience and nanotechnology is helping nanotechnologists to cope with the challenges of water treatment. Different alternatives like zero-dimensional nanoparticles,^3^ one-dimensional nanowire/nanorods,^4,5^ two-dimensional nanosheets,^6,7^ three-dimensional nanostructures^8^ along with their different functional composites,^9–11^ are helping the researchers in bringing down wastewater treatment dilemma.

As, the two dimensional (2D) materials have manifested excellent outcomes in the field of adsorption, graphene and its derivative due to their intriguing physical and chemical properties^12–14^ and specific characteristic properties, particularly their size, structure, density, and porosity have shown the marvelous performance. The graphene specifically has attracted researcher's interest in soliciting it in wide varieties of applications in nanotechnology to tackle water treatment problems.^15,16^ The material scientists are fabricating functionalized nanoporous graphene-based composites.^17–19^ The top-down exfoliation has led to excellent yield of 2D materials in an affordable way, due to the trouble free accessibility of layered crystals *i.e.* graphite.^20^

Various methods have been developed for the synthesis of 2D nanoporous materials such as energetic particle bombardment^21–25^ and chemical reduction such as chemical etching (CE), oxygen gas etching (OGE), ultra violet-induced oxidative etching (UIOE), oxygen plasma (OP) etching, electrical pulse (EP) method and electrochemical reaction (ECR) *etc.* However, it is quite difficult to elaborate a universal description of these methods due to their complex reaction mechanisms.^26–31^

Therefore, the alternative for these methods includes the method of radiation induced reduction that is a non-chemical patterned approach and leads to the feasible formation of porous graphene. Various microwave assisted methods are gaining keen interest of researchers for synthesis and modification of graphene and its derivatives^32,33^ due to their simplicity, cost effectiveness and swiftness. Recently, James Laurence *et al.*^34^ reported microwave-induced fabrication of fiber-reinforced adsorbent from waste cardboard and chitosan for the removal of Congo red. The material showed adsorption capacity of 53.87 mg g^−1^ for Congo red. Similarly, Araichimani *et al.*^35^ reported microwave assisted synthesis of Fe_3_O_4_-decorated SiO_2_ nanostructure for the removal of Hg^2+^ ions from aqueous environment. The material showed more than 90% removal of Hg^2+^ ions at pH 6. Hijab *et al.*^36^ reported fabrication of date-stone activated carbon using microwave and thermal methods. The results revealed that activated carbon obtained from microwave method had greater surface area (1123 m^2^ g^−1^) than activated carbon obtained using thermal method (669 m^2^ g^−1^). Both activated carbons were utilized for the removal of malachite green and maximum malachite green adsorption capacities were found as 58 mg g^−1^ and 98 mg g^−1^ for thermally activated carbon and microwave-assisted activated carbon, respectively. Here, it is worth mentioning that most of the reported adsorbents are capable of removing one or two toxins at a time; however, there is an urgent need to develop a universal adsorbent that has capacity to remove maximum contaminants from aqueous system.

The above discussion elaborates that the microwave treatment leads to the materials having high surface area and adsorption capacity. It is also helpful for substantial reduction and expansion of the GO simultaneously and is responsible for its porous morphology that evince its suitability for adsorption purpose as well. It is because the defects produced on nanosheets serve as active sites that are fruitful for the adsorption of contaminants.

In the current study we have synthesized novel GO based nanoporous material with tuned hydrophilicity by microwave radiation, that shows excellent removal efficiency towards the organic pollutants as well as toxic metal ions in the industrial wastewater. GO is hydrophilic in nature, as well as produces high back pressure when used as column material for removal of contaminants. Therefore, vacuum filtration is applied for water purification that ultimately increases the cost of purification procedure. Moreover, capacity of pure GO is also very limited for contaminants due to its limited surface area.

Therefore, the main objective of current research study was to develop a graphene oxide-based composite (GOBC) material with high selectivity, capacity, and efficiency for removing BPA, BPS, endosulfan, beta-estradiol, dyes and toxic metal ions from wastewater. For this purpose, GO was first intercalated with 1-octanesulphonic acid sodium salt. The major reason for choosing 1-octanesulphonic acid sodium salt was its hydrocarbon chain, sulphonic acid group and its ability to allow ion exchange phenomenon due to the presence of sodium ions. The long hydrocarbon chain introduced hydrophobic moieties into the resulting GOBC and sulphonic acid group facilitated the absorption of microwaves radiation and ultimately simultaneous reduction and expansion of subjected GO along with production of nanopores. A fixed bed (FB) column study and Jar-test study was carried to determine the adsorption capacity. Real water samples (before/after adsorption) were analyzed through GC-MS and ICP-OES to counter check the better performance of synthesized material.

## 2. Experimental

### 2.1. Materials

Bisphenol S, bisphenol A, methylene violet, methylene blue, 1-octanesulphonic acid sodium salt, graphite flakes, sodium nitrate (99.0%), hydrochloric acid, sulphuric acid (35%), potassium permanganate (99.0%), hydrogen peroxide (35%), methanol (99.9%), cadmium nitrate, sodium arsenate, copper nitrate, lead nitrate, nickel sulphate, cobalt chloride, mercury(ii) acetate, zinc nitrate and lithium chloride were purchased from Sigma-Aldrich, USA. Analytical grade reagents were used throughout the entire study. De-ionized water was used for all studies.

### 2.2. Synthesis of graphene oxide based composite

By reported modified Hummer's method GO was synthesized from graphite flakes.^37^ For the modification of GO, 0.1 g of GO was dispersed in 20 mL of DI water by ultra-sonication. Intercalation of well dispersed GO was carried by adding 0.06 g of 1-octanesulphonic acid sodium salt into the dispersed GO and mixture was further ultra-sonicated for 30 min. After intercalation, mixture was subjected to the microwave radiation for 30 min in 60 cycles (200 watt, 30 s on and 1 min off). After microwave treatment the prepared material was washed under centrifuge, dried and then again subjected to microwave treatment at 800 watts (10 cycles, 30 s on and 1 min off). The resulting material was highly expanded nanoporous GOBC.

### 2.3. Characterization

X-Ray Diffractometer (XRD) model XRD-700-Shimadzu was used to investigate the crystalline nature of prepared GO and composite material. The surface morphology of prepared material was characterized by scanning electron microscope (SEM) model SEM-JSM 7800F, Fourier transform infrared spectroscopy (FTIR) (Nicolet-5700, Themofinnigan, USA) was used to determine functionalities and UV-visible spectroscopy (UV-vis) (Lambda-35, PerkinElmer, USA) was used to determine the concentration of analytes before/after adsorption. Zeta potential model DLS (sizing), M3-PALS (zeta potential) was used to determine the charge on surface of material. BET (AutosorbiQ S/N: 14716090801 station: 1) analysis was used to check the surface area and pore diameter of prepared material. Raman analysis was performed using (DXR Raman microscope with a 780 nm filter, Thermo Scientific). The contact angle of the prepared materials was estimated using a standard contact angle apparatus (Ossila contact Angle Goniometer). Metals analysis was performed by ICP-OES model Thermo Scientific iCAP 7000 spectrophotometer. The GC/MS analysis was carried out using an Agilent 6900 gas chromatograph and an Agilent 5975 mass spectrometer (Agilent Technologies, US).

### 2.4. Jar-test experiments

Adsorption experiments were also performed using Jar-test. Precisely, the BPA was adsorbed on various mass concentrations of GOBC such as 0.2, 0.4, 0.6 and 1.0 g. The Jar-test was performed using 1 L beakers assembled with mechanical stirrer adjusted at rotation of 50 rpm, with a contact time of 2 h at ambient temperature. The BPA solution of 0.22 mmol L^−1^ (50 mg L^−1^) was adsorbed using GOBC of different amounts.^38^ After completion of adsorption process the final concentration of BPA was analyzed using UV-Visible spectrophotometer. The concentration of BPA adsorbed per gram of GOBC was calculated using eqn (1).1
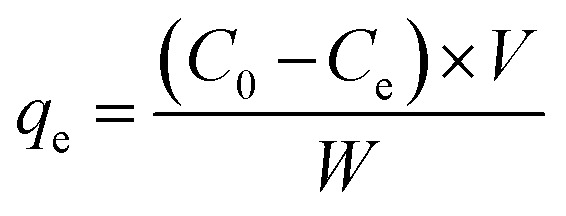
where *W* is mass of adsorbent (g), *V* is the volume of the solution (L), *C*_0_ and *C*_e_ are the initial (before adsorption) and final (after adsorption) concentrations of BPA, respectively (mg L^−1^). The *q*_e_ is the adsorption capacity at equilibrium per mass of GOBC (mg g^−1^). The optimized amount of GOBC was utilized to estimate the maximum adsorption time for BPA. After optimizing the suitable amount and adsorption time of GOBC; different concentrations of BPA were adsorbed on the optimized amount of GOBC to estimate its maximum adsorption capacity.

The obtained experimental data was further analyzed using Langmuir (1918) (eqn (2))^39^ and Freundlich (1906) (eqn (4))^40^ mathematical isotherms to understand the characteristics of adsorption. The pseudo-first order kinetic model proposed by Lagergren (1898) (eqn (5)),^41^ and pseudo-second order kinetic model proposed by Ho and McKay (1998) (eqn (6))^42^ were used to elaborate the kinetic studies.2
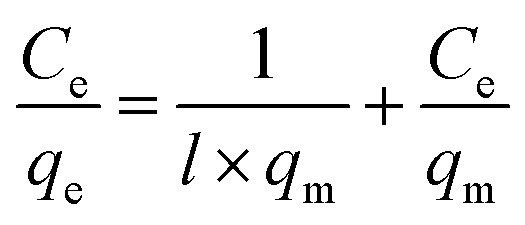
where *q*_m_ is the maximum amount of BPA adsorbed per mass of adsorbent at equilibrium (mg g^−1^) and *l* is the Langmuir constant related to the affinity of the adsorbent and adsorbate (L g^−1^).

The Langmuir separation factor (*R*_l_) was also calculated, using the eqn (3) proposed by McKay *et al.* (1982).^43^3
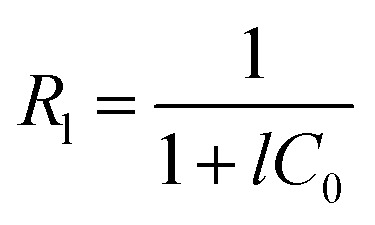
4
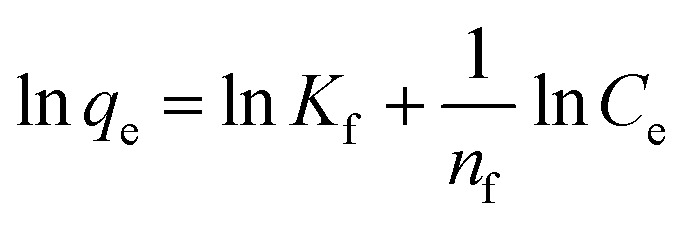
where *K*_f_ is the Freundlich constant related with solid adsorption capacity (L g^−1^), and *n*_f_ is the adsorption constant. Eqn (4) represents the linear Freundlich model and the linear regression plot of ln *q*_e_*vs.* ln *C*_e_ allows the calculation of *K*_f_ and *n*_f_ values.

5 log(*q*_e_ − *q*_*t*_) = log(*q*_e_) − (*k*_1_*t*)/2.303

where *q*_e_ is the amount of BPA adsorbed at equilibrium (μmol g^−1^) and *q*_*t*_ is the amount adsorbed at time *t*, respectively, and *k*_1_ denotes the rate constant of the pseudo first order kinetic model (min^−1^).6
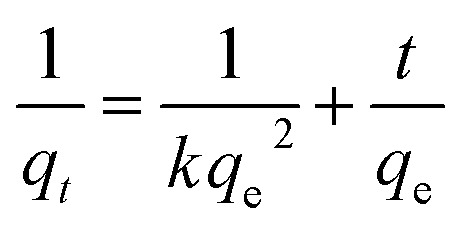
where *q*_e_ and *q*_*t*_ have the same meaning as in eqn (5), and *k* is the rate constant of pseudo second order kinetic model (g μmol^−1^ min^−1^).

### 2.5. Fixed-bed column experiment

Fixed-bed column experiment was used to check the removal effectiveness of the prepared material for BPA. The column was prepared in cartridges having 2 cm diameter and 5 cm length. A known amount of an adsorbent was packed inside the column between supporting layers of frits. For the breakthrough curve analysis various parameters were optimized such as effect of bed height, flow rates and adsorbate concentrations. Precisely, in order to optimize bed height, different bed heights in the range of 0.1 to 0.4 cm were used. To optimize flow rate, experiments were performed by changing flow rate in the range of 0.5 to 1 mL and feed concentration was varied in the range of 200 to 300 ppm. Downward flow mode was used throughout the column study. As the study focused on the removal of contaminants from real water samples, all experiments were performed at ambient conditions. The solid phase extraction assembly was used in the experiment as an extractor. At various time periods effluent solution was collected for the examination of exhaustion point and breakthrough point for plotting breakthrough curve and the tests were carried out until the column was completely saturated. Effluent concentration was analyzed by UV-vis Spectrophotometer, GCMS and ICPOES. Mathematical kinetic models such as Adam–Bohart, Yoon–Nelson and Thomas were used to fit experimental adsorption data. Breakthrough curves data, adsorption process with bed heights, feed concentrations and flow rates were evaluated.

## 3. Results and discussion

### 3.1. Synthesis of GOBC

**Fig. 1:**
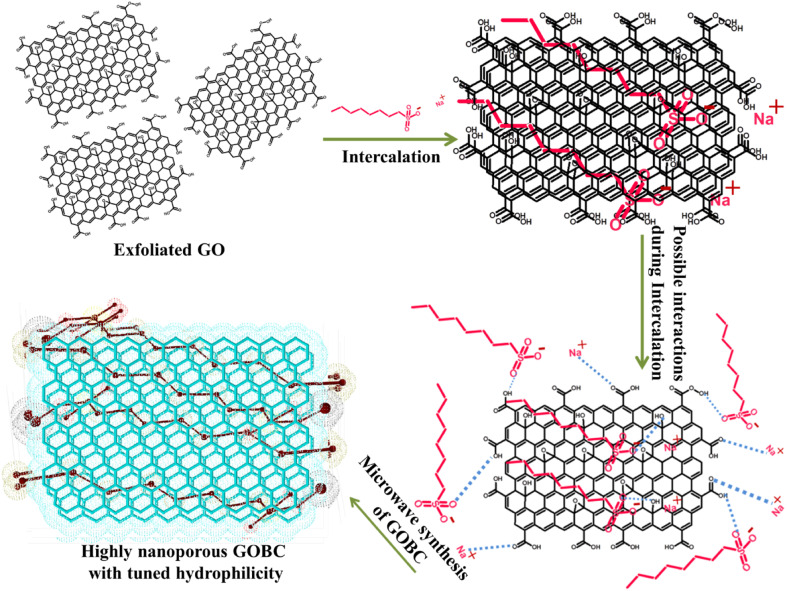
Schematic representation of fabrication of GOBC.

The presence of oxygen containing functionalities such as carboxylic acid, hydroxyl groups, epoxy and alkoxy *etc.* makes GO extremely hydrophilic. Thus, GO can effectively adsorb hydrophilic contaminants from aqueous environment and shows poor adsorption for hydrophobic contaminants. Moreover, the surface area of GO is also very low due to the presence of limited number of nanopores in its structure.^44^ Therefore, GOBC was aimed to possess tuned hydrophilicity so that it could adsorb both highly hydrophilic and less hydrophilic organic contaminants from the industrial wastewater, effectively. Moreover, it should effectively adsorb the toxic metal ions from aqueous environment and possess high surface area with extremely nanoporous structure. For this purpose, 1-octane sulphonic acid sodium salt was first intercalated into the layers of GO. The intercalation occurred due to the interaction of sulphonic acid functionality in the 1-octane sulphonic acid sodium salt with hydroxyl functionalities of GO *via* hydrogen bonding.^45^ Later, the resulting GO was subjected to reduction *via* microwaves. Here, it is noteworthy that GO remains in active and does not irradiate by microwaves. In order to accomplish microwave assisted reduction of GO there is need to introduce such functionalities in its structure that can conduct microwaves.^46^ The compounds containing sulphur such as sodium hydrogen sulfite (NaHSO_3_), sodium sulfide nonahydrate (Na_2_S·9H_2_O), sodium thiosulfate (Na_2_S_2_O_3_), and thionyl chloride (SOCl_2_), have already been used to reduce GO by decreasing the concentration of hydroxyl and epoxide groups.^47^ Hence, 1-octane sulphonic acid sodium salt intercalated into the layers of GO allowed the absorption of microwaves due to the presence of sulphonic acid functionality in its structure, the process led to the reduction of GO by removing oxygen functionalities. The microwave treatment of 1-octane sulphonic acid sodium salt intercalated GO not only reduced GO extensively but also converted the resulting composite into highly expanded and porous material. The resulting GOBC could effectively adsorb hydrophilic organic contaminants due to the presence of sulphonic acid functionality, slightly hydrophobic compounds due to the presence of long hydrocarbon chain and toxic metal ions due to the presence of nanopores and ion exchange capacity into its structure.^48^ The 1-octane sulphonic acid sodium salt had been successfully utilized as mobile phase for HPLC based separations due to its ability to exchange ions and lead separation.^49^ Hoa Ren *et al.*^50^ also reported GO–BT composite for effective removal of Phenols. Fig. 1 shows schematic representation of fabrication of GOBC. GOBC due to its excellent structural composition is capable of removing several organic contaminants and toxic metal ions. Also, the obtained adsorption capacities are superior than the adsorption capacities of reported adsorbents.

### 3.2. Characterization

**Fig. 2:**
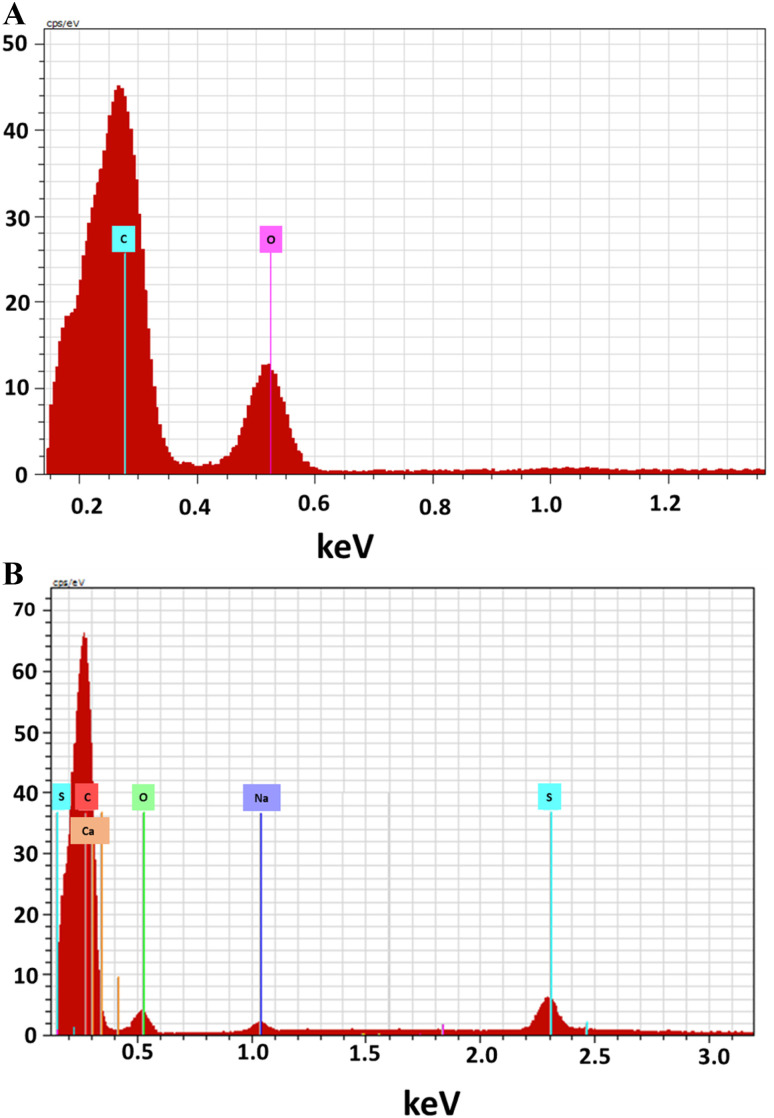
EDX analysis of (A) GO and (B) GOBC.

EDX study was used to examine the composition of prepared materials. The EDX spectra of GO (Fig. 2A) revealed it is composed of carbon (C, 73.64%) and oxygen (O, 26.36%); the spectra further confirmed that graphite was successfully oxidized into GO. However, in EDX spectrum of GOBC (Fig. 2B) along with carbon (C, 89.69%) and oxygen (O, 9.84%); sulfur (S, 0.71%) and sodium (Na, 0.37%) are also present. These results confirmed that interlayers of GO were successfully intercalated with 1-octanesulphonic acid sodium salt. Furthermore, after the microwave treatment the oxygen percentage has also been reduced from 26.36 to 9.84% that confirmed the successful fabrication of reduced GO which is well modified with 1-octanesulphonic acid sodium salt. The results obtained from EDX confirmed that elemental composition of GOBC is well organized in order to make it compatible for efficient removal of selected EDCs.

**Fig. 3:**
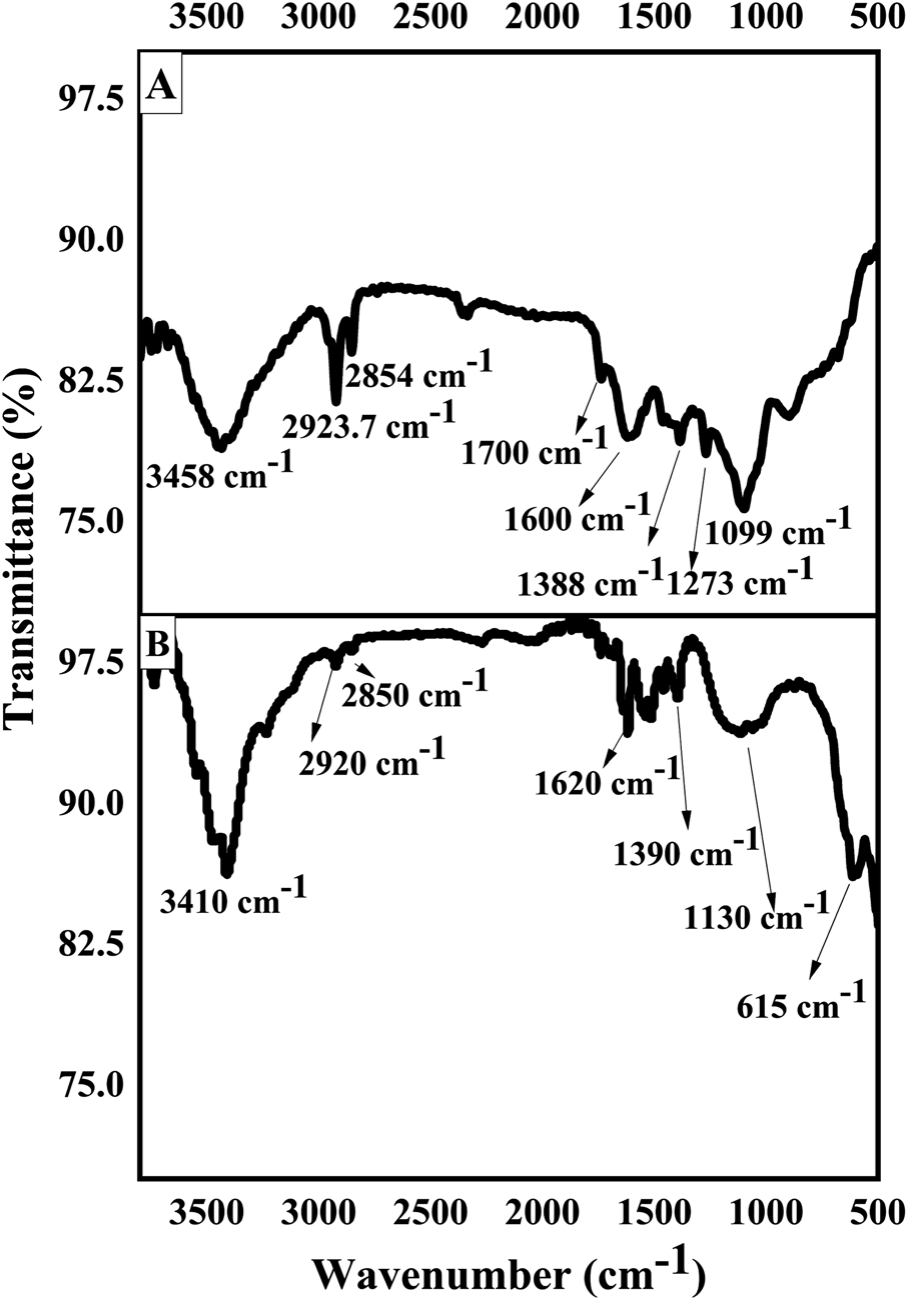
FTIR spectra of (A) GO and (B) GOBC.

FTIR is used to identify presence of different functionalities in the fabricated materials.^51^ As revealed in (Fig. 3A) the FTIR spectra of GO exhibits several peaks showing oxygen-containing functionalities, indicating that graphite was successfully oxidized. The wide peak at 3458 cm^−1^ is attributed to –OH stretching. The peaks at 2923.7 cm^−1^ and 2854 cm^−1^ correspond to the asymmetric and symmetric stretching vibrations due to –CH_2_. The peak at 1600 cm^−1^ is due to stretching vibration of the (C=C) and the deformation of C–O peak is observed at 1388 cm^−1^.^52^ The peaks present at 1700 cm^−1^, 1273 cm^−1^ and 1099 cm^−1^ confirmed the presence of carbonyl, epoxy, and alkoxy functional moieties, respectively.^53^ The FTIR spectrum of GOBC (Fig. 3B) shows a broad peak at 3410 cm^−1^ due to –OH stretching vibration from sulphonic acid functionality. The peaks at 2920 cm^−1^ and 2850 cm^−1^ due to asymmetric and symmetric stretching vibrations of –CH_2_ functionality have appeared with weaker intensity as compared to GO. The characteristic peaks due to carbonyl, epoxy, and alkoxy functional moieties have been disappeared in GOBC that confirmed the reduction of GO. The stretching vibration due to C=C has been shifted to 1620 cm^−1^ in GOBC. Furthermore, the new peak appeared at 1390 cm^−1^ is due to the –CH_3_ stretching vibration from octane group and peaks at 1130 cm^−1^ and 615 cm^−1^ are due to the S–O stretching vibrations from sulphonic acid group, respectively.^54^ The obtained FTIR results are in line with results obtained from EDX. Further, it is confirmed that GOBC is functionalized successfully and is capable to interact with selected toxins effectively.^48,55^ The results obtained from IR spectroscopy are summarized in Table 1.

**Table:** Details of FTIR vibrations of GO and GOBC

GO		GOBC	
Wavenumber (cm^−1^)	Functionality	Wavenumber (cm^−1^)	Functionality
3458	–OH stretching	3410	–OH stretching
2923.7	–CH_2_ asymmetric	2920	–CH_2_ asymmetric
2854	–CH_2_ symmetric	2850	–CH_2_ symmetric
1700	–C–O stretching	1620	C=C stretching
1600	C=C stretching	1390	–CH_3_ functionality
1388	C–O deformation	1130	S–O stretching
1273	Epoxy functionality	615	S–O stretching
1099	Alkoxy functionality		

**Fig. 4:**
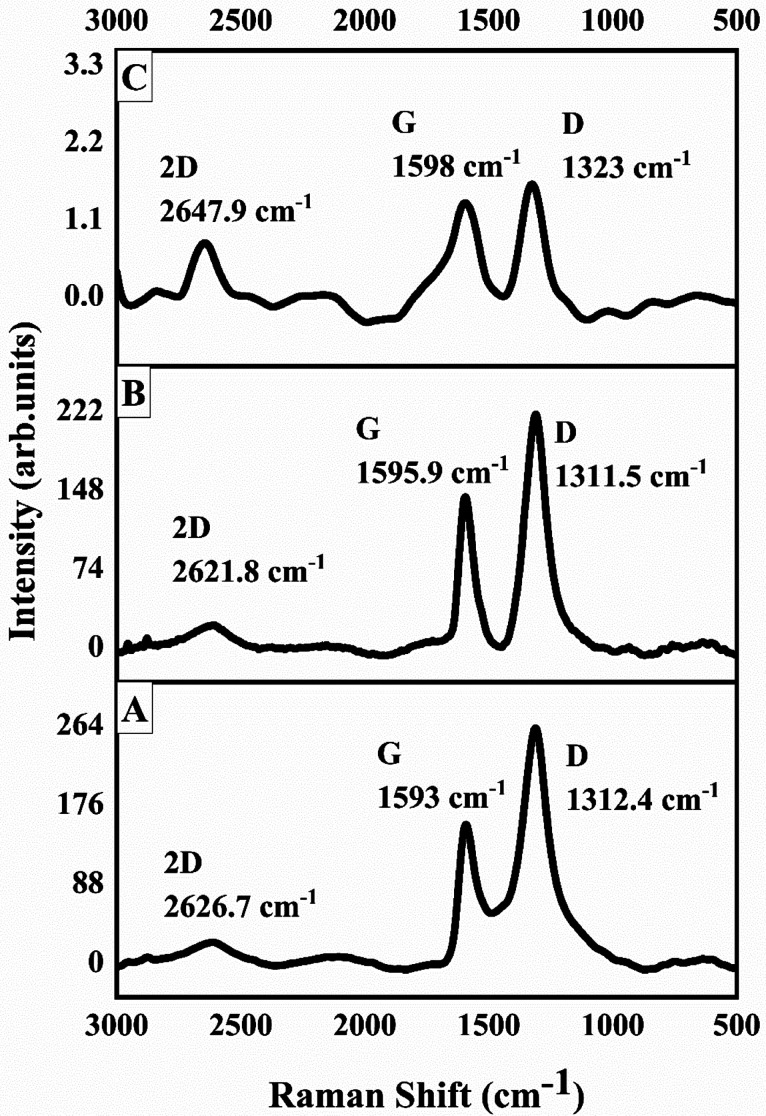
Raman spectra of (A) GO, (B) 1-octane sulphonic acid sodium salt intercalated GO and (C) GOBC.

Raman spectroscopy is an important tool to investigate carbon materials because of the fact that conjugated and carbon–carbon double bonds show high Raman intensities. Graphite is highly ordered carbon material and shows few Raman-active bands. The prominent G band appears at around 1575 cm^−1^ due to in-phase vibration of graphite lattice and a weak D band also known as disorder band appears around 1355 cm^−1^ due to graphite edges.^56^ The D band in graphite remains inactive until it loses its symmetry; the loss in symmetry causes the edges to produce dominant D band. Both the G and D bands undergo prominent changes upon modification and amorphization of graphitic materials.^57^ Broadly, the prominent disorder in graphite corresponds to a broader G and D bands with higher relative intensity of D band as compared to G band. Fig. 4A shows Raman spectrum of GO. The prominent and broad G and D bands appear at 1593 cm^−1^ and 1312.4 cm^−1^, respectively. The intensity of D band is also higher than G band, that confirmed the extent of disorder caused in graphitic sheets due to oxygen containing functionalities such as hydroxyl and epoxy groups. A weak 2D band at around 2626.7 cm^−1^ also appeared. The shape of 2D band and position of G band are helpful in estimating number of graphitic sheets stacked. Fig. 4B is the Raman spectra of 1-octane sulphonic acid sodium salt intercalated GO. The results revealed that G band in 1-octane sulphonic acid sodium salt intercalated GO shifted to the higher frequency from 1593 cm^−1^ (GO) to 1595.9 cm^−1^ which indicates further amorphization of GO. The D and 2D bands are appeared at 1311.5 cm^−1^ and 2621.8 cm^−1^, respectively.^58^Fig. 4C shows the Raman spectrum of GOBC; here the intensities of G and D bands have been reduced greatly that confirmed the formation of single layer graphene composite. Moreover, the decrease in the intensity of G band (1598 cm^−1^) also confirmed the formation of single layer graphene composite. The single symmetric peak of 2D band at 2647.9 cm^−1^ in GOBC also confirmed the presence of single layer graphene.^59^

**Fig. 5:**
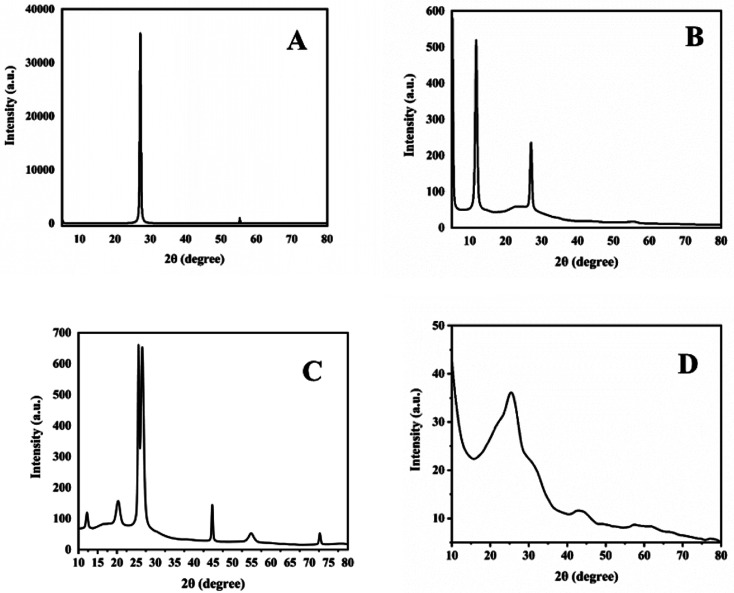
XRD spectra of (A) graphite, (B) GO, (C) 1-octane sulphonic acid sodium salt intercalated GO and (D) GOBC.

XRD analysis is used to check the crystallographic structure of materials and their chemical composition information. XRD patterns of graphite, GO, 1-octane sulphonic acid sodium salt intercalated GO and GOBC are shown in (Fig. 5). The XRD pattern of graphite (Fig. 5A) showed sharp and intense diffraction peak at 2theta 27° with *d*-spacing of 0.33 nm which reflects highly crystalline nature of graphite. Well in case of GO (Fig. 5B) there is appearance of new peak at 2theta 11.7° due to the presence of oxygenated sheets of GO; having *d*-spacing of 0.75 nm which is higher than graphite. It is due to the presence of oxygenated moieties on the surface of GO layers because of the extensive oxidation that produced repulsive forces between sheets. The peak at 2theta 27° is due to the presence of non-oxygenated sheets of graphite. However, the decrease in intensity of peak at 2theta 27° indicated decrease in the crystalline nature of graphitic sheets.^60^Fig. 5C represents the XRD pattern of 1-octanesulphonic acid sodium salt intercalated GO. The diffractogram clearly indicated the structural changes in GO after intercalation. For instance, the intensity of peak due to oxygenated sheets at 2*θ* 12.2° has been greatly reduced that revealed the reduction of GO. Furthermore, appearance of peaks at 2*θ* 20.29°, 25.58°, 44.74°, 54.84° and 72.67° indicated the monolayer assembly of 1-octanesulphonic acid sodium salt into the layers of GO.^61^ Similar results were presented by Thomas Arnold *et al.*^62^ when they adsorbed even number alkanes on the surface of graphite. However, there are minute shifts observed in 2*θ* due to the presence of sulphonic acid and sodium metal. The sulphonic acid based sodium salts also produce XRD patterns in almost similar region.^63^ Furthermore, the reappearance of peak at 2*θ* 26.57° with *d*-spacing of 0.335 further confirmed the reduction of GO during intercalation process. The reduced intensity of peak at 2*θ* 26.57° as compared to graphite confirmed the loss in crystallinity of graphite with sheet height of 8.6 nm as compared to sheet height of graphite (21.6 nm). The *d*-spacing due to the oxygenation of graphite into GO was found as 0.753 nm that was reduced to 0.44 nm (calculated from peak at 2*θ* 20.29°) in the 1-octanesulphonic acid sodium salt intercalated GO. After the intercalation with 1-octanesulphonic acid sodium salt and microwave treatment at 800 watt, the disappearance of peak at 2theta 11.7° occurred that confirmed the reduction of oxygenated functionalities and the broad peak with reduced intensity at 2theta 27° confirmed that the crystalline nature of material is decreased and GOBC is converted into nano-graphene with sheet height of 5.3 nm.^64^ Here, it is worth mentioning that the intercalating precursor 1-octanesulphonic acid sodium salt allowed GO to absorb microwave radiations so that GO could reduce; moreover, it remained intercalated even after microwave treatment and produced reduced graphene oxide with tuned hydrophilicity. The results of XRD are in good agreement with results of EDX.

**Fig. 6:**
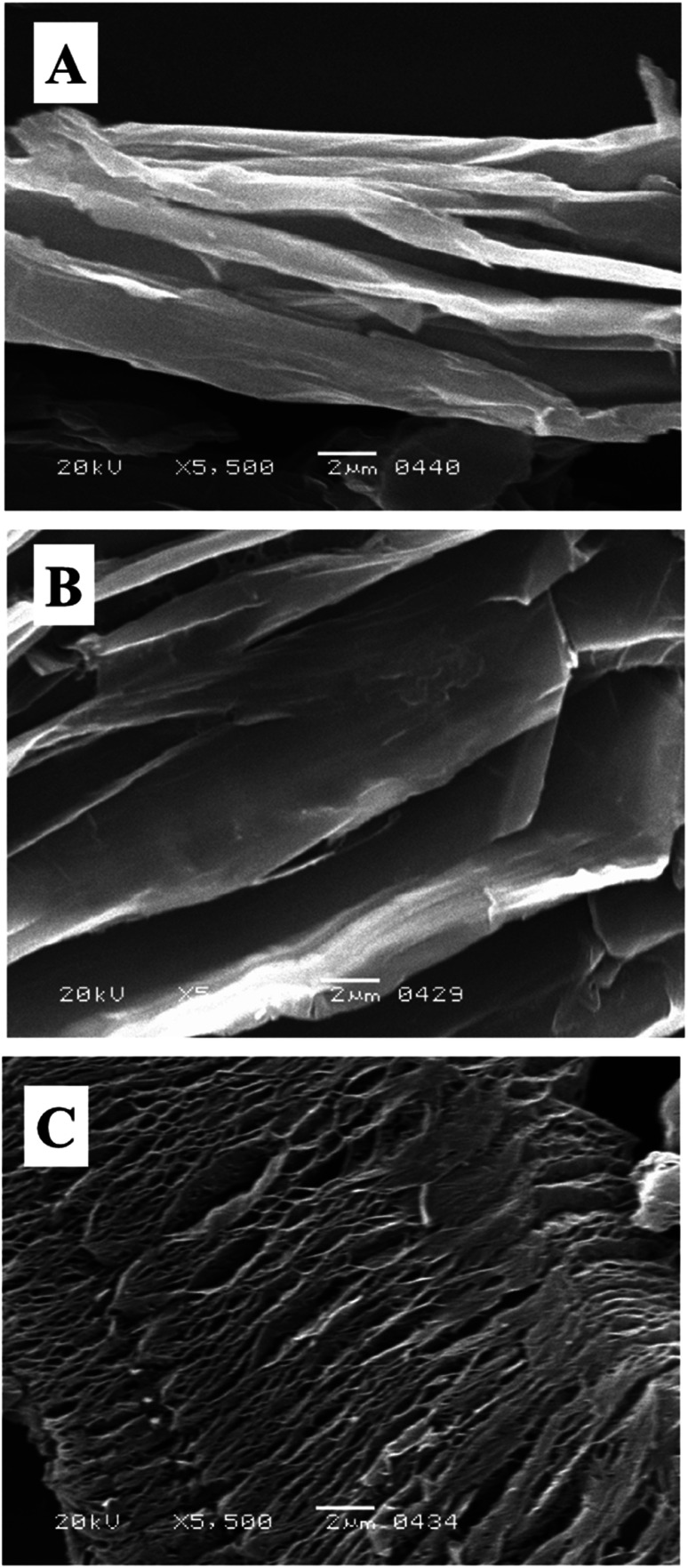
SEM micrograph of (A) GO, (B) intercalated GO and (C) GOBC.

SEM study is used to investigate the surface morphology of composite material.^65^ As shown in Fig. 6A that the graphite is exfoliated successfully to GO. From the SEM micrograph the inter layer distance is quite visible and indicated that graphite is well oxygenated, and highly crystalline in nature.^66^Fig. 6B shows the SEM micrograph of intercalated GO; here, we observed that after intercalation the inter layer distance between sheets further increased. Moreover, the sheets are thinner than GO that indicated lesser number of stacked sheets in intercalated GO as compared to GO and confirmed further exfoliation. After the microwave treatment it could be seen clearly that the sheets have expanded very well (Fig. 6C). It is because of the presence of sulphonic acid functionality into the layers of GO which is facilitating GO to conduct microwaves. The efficient absorption of microwaves resulted in highly expanded GOBC.^67^ The successfully expanded graphene-based materials are proved to be excellent adsorbents for removal of contaminants from aqueous environment using fixed-bed column method because these materials allow the passage of contaminated aqueous fluids easily and have greater reusability.^68^

**Fig. 7:**
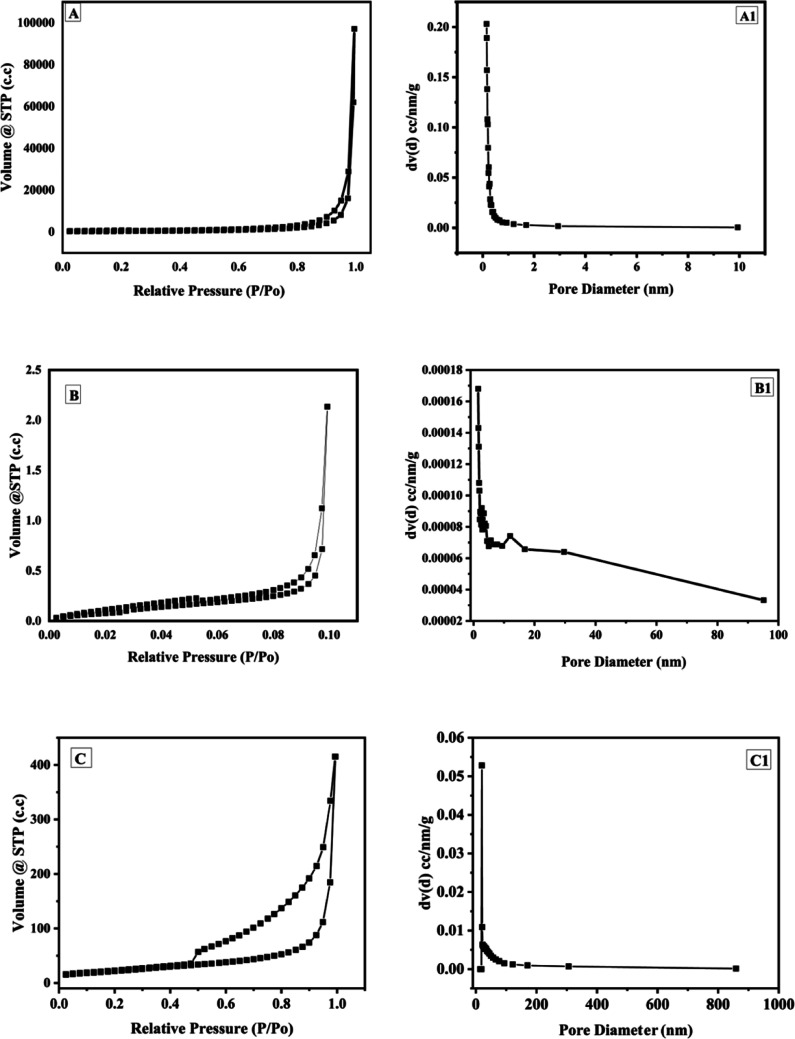
BET isotherm of (A) graphite, (B) GO and (C) GOBC; BJH adsorption isotherm of (A1) graphite, (B1) GO and (C1) GOBC.

The BET analysis is shown in Fig. 7. The BET and BJH adsorption isotherms of graphite (Fig. 7A), GO (Fig. 7B) and GOBC (Fig. 7C) suggested that the surface area of GOBC has drastically increased in comparison to GO and graphite, it increased from 3 m^2^ g^−1^ (graphite) to 80 m^2^ g^−1^ (GOBC). Further, the pore volume of GOBC (0.628 cm^3^ g^−1^) was 95% more than the pore volume of graphite (0.033 cm^3^ g^−1^) that confirmed fabrication of highly porous GOBC after microwave treatment. The fabricated GOBC expanded very well and the pore diameter was smaller (1.7 nm) than the pore diameter of GO (1.9 nm). However, it was higher than graphite (1.5 nm) as shown in Table 2. The BET results revealed that applied microwave-based synthesis strategy is excellent for producing graphene derivatives with tuned properties and highly enhanced surface area. Furthermore, the expansion of GO did not compromise the pore size of graphene sheets and resulting GOBC is highly porous material with controlled nanopores size and extremely high surface area. The BET isotherms of graphite, GO and GOBC showed the formation of hysteresis loops as the desorption path is different from the adsorption path. These hysteresis loops confirmed the occurrence of capillary condensation in the mesopores of materials.^69^ The type of hysteresis loop gives the information about pore shape. The BET isotherms showed that graphite, GO and GOBC follow hysteresis loop of type 3 (H3). The Loops of type H3 are found in solids consisting of aggregated non-rigid platelike particles such as 2D materials like graphene and its derivatives.^70^ The BET results provided further prove that GOBC is an eligible broad-spectrum candidate for water treatment because of its drastically enhanced surface area and increased volume of nanopores. The improved nanoporous morphology of GOBC plays an important role in removal of toxic metal ions.^71^

**Table:** BET analysis of graphite, GO and GOBC

Name	MBET surface area, m^2^ g^−1^	Pore volume, cm^3^ g^−1^	Pore diameter *D*_v_(*d*), nm
Graphite	3.11	0.033	1.5
GO	1.52	0.002	1.9
GOBC	80	0.628	1.7

**Fig. 8:**
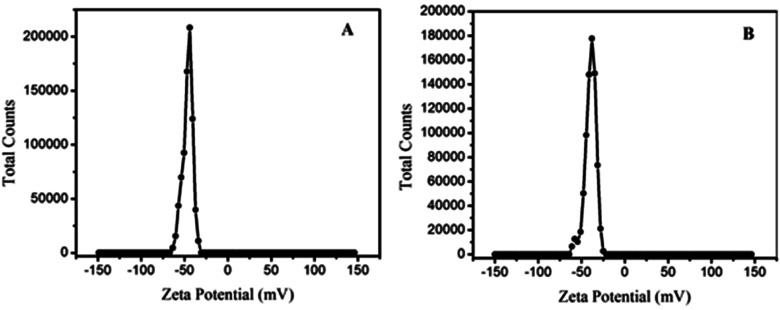
Zeta potential charge distribution of (A) GO and (B) GOBC.

Zeta potential charge distribution was carried out to know the surface charge of the materials (Fig. 8). The obtained zeta potential charge distribution on GO was −46.5 mV; however, the charge distribution of GOBC was obtained as −36 mV. The zeta charge distribution data showed that both GO and GOBC have negative surface charge, and zeta potential charge distribution confirmed that material is highly stable and it resists the agglomeration of sheets. Furthermore, the surface charge of GOBC is higher than GO which is due to the reduction of GO and presence of hydrocarbon chains and reduced graphene planes in the structure of GOBC. Precisely, the exceptional surface functionalities of GOBC such as presence of long hydrocarbon chain, sulfonic acid group, ion exchange capacity and its nanoporous expanded sheets morphology are making GOBC an outstanding material for the removal of many contaminants having variety of functional properties.

**Fig. 9:**
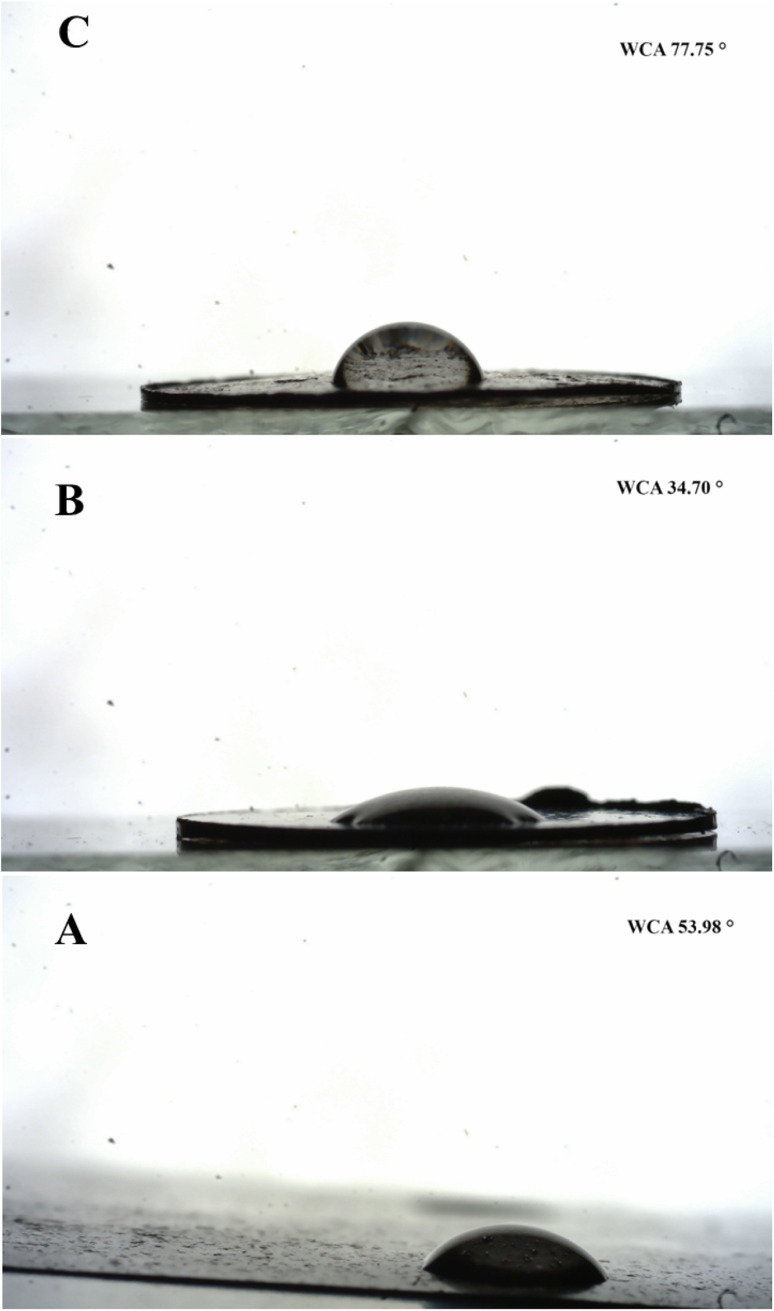
Contact angle of (A) GO, (B) 1-octane sulphonic acid sodium salt intercalated GO and (C) GOBC.

The wettability of adsorbent is an important characteristic that defines its effectiveness for the adsorption of hydrophilic or lipophilic contaminants. Therefore, the wettability of GO, 1-octane sulphonic acid sodium salt intercalated GO and GOBC surfaces was examined by stationary water contact angle. The water contact angle of GO was found as 53.98° due to the presence of hydrophilic oxygen containing functional groups. The water contact angle greatly decreased as expected in the case of 1-octane sulphonic acid sodium salt intercalated GO (34.70°) due to the addition of sulphonic acid functionality which enhanced its hydrophilicity.^72^ The water contact angle of GOBC was obtained as 77.75°. The drastic increase in water contact angle from 34.70° to 77.75° (Fig. 9) was due to the successful reduction and removal of oxygen containing functionalities *via* microwave treatment. GOBC with tuned wettability proved itself more efficient adsorbent as compared to GO.^73^

## 4. Adsorption studies

### 4.1. Jar-test adsorption studies

**Fig. 10:**
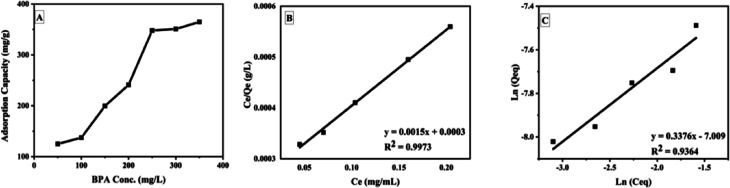
Plot of (A) adsorption capacity of GOBC, (B) Langmuir isotherm and (C) Freundlich isotherm.

The Jar-test adsorption studies showed that GOBC was settled at the bottom of the beakers after suspension of stirring. However, during the adsorption process under mild stirring GOBC remained suspended in the solution; hence GOBC would be a feasible adsorbent for the upscaled industrial applications. The GOBC showed an adsorption capacity of 350 mg g^−1^ at ambient conditions. Here, it is noteworthy that all the adsorption experiments were performed without adjusting pH (at neutral pH) that makes GOBC cost effective as pH adjustment of treatment tank is not required before and after treatment. Carolina Rosai Mendes *et al.*^38^ also reported Jar-test adsorption for the removal of industrial dye DO 2GL using chitosan-based biopolymer. The Jar-test adsorption studies showed the adsorption capacity of biopolymer as 24.615 mg g^−1^ at pH 6.5. They also claimed that adjustment of treatment tank output is not required as the adsorption pH is near to neutral. However, the pH adjustment was required for the effective adsorption of DO 2GL. Fig. 10 shows the plot of adsorption capacity of GOBC *vs.* concentration of BPA and plots of Langmuir and Freundlich isotherms. The plot of adsorption capacity (Fig. 10A) reveals that the adsorption capacity of GOBC is increasing with the increasing concentration of BPA until it reaches its maximum adsorption capacity. The isotherms study of adsorption test (Table 3) confirmed that the GOBC's adsorption fits very well to the Langmuir adsorption isotherm (Fig. 10B) which indicates that the GOBC follows site specific adsorption of the analyte and forms monolayer. The value of *l* indicates that GOBC has excellent adsorbate/adsorbent affinity and results were further confirmed by calculating *R*_l_ value (0.0015) that approaches 0 and therefore, indicates that adsorption is nearly irreversible. The obtained results indicated the chemical interaction between adsorbent and adsorbate.

Adsorption isotherms parameters and kinetics models parameters for the adsorption of BPA on GOBC

**Table:** 

Langmuir isotherm					Freundlich isotherm			
*q*_m_ (L g^−1^)	*l* (L g^−1^)	*R*_l_	*R* ^2^	SD	*K*_f_ (L g^−1^)	*n*_f_	*R* ^2^	SD
666.7	5.0	0.0015	0.9971	1.11	1106.5	2.96	0.936	2.63

**Table:** 

Pseudo first order					Pseudo second order			
*q*_e_ (exp) (mg g^−1^)	*k*_l_ (min^−1^)	*q*_e_ (cal) (mg g^−1^)	*R* ^2^	SD	*k*_s_ (g mg^−1^ min^−1^)	*q*_e_ (cal) (mg g^−1^)	*R* ^2^	SD
125	0.0006	4.23	0.813	2.54	0.0032	128	0.9986	0.432

**Fig. 11:**
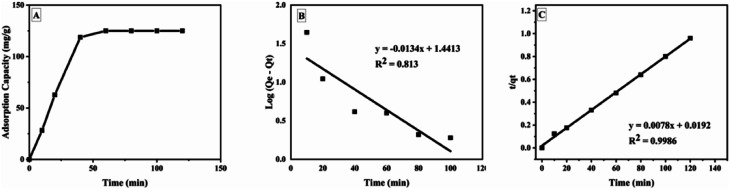
Plot of (A) time study, (B) pseudo first order kinetic model and (C) pseudo second order kinetic model.

The kinetic studies showed that GOBC reached the adsorption equilibrium at around 40 min. The adsorption pattern of GOBC correlates well with the pseudo second order kinetic model (Table 3) as the *q*_e_ (cal) (128 mg g^−1^) obtained from pseudo second order kinetic model is very close to the *q*_e_ (exp) (125 mg g^−1^). These results further confirmed that adsorption occurs *via* chemical interactions as the adsorbents following pseudo second order kinetic model follow adsorption through chemical process.^74^Fig. 11 shows the time study and kinetic models for the adsorption of BPA on GOBC.

### 4.2. Column adsorption studies

#### 4.2.1. Effect of bed heights on adsorption

**Fig. 12:**
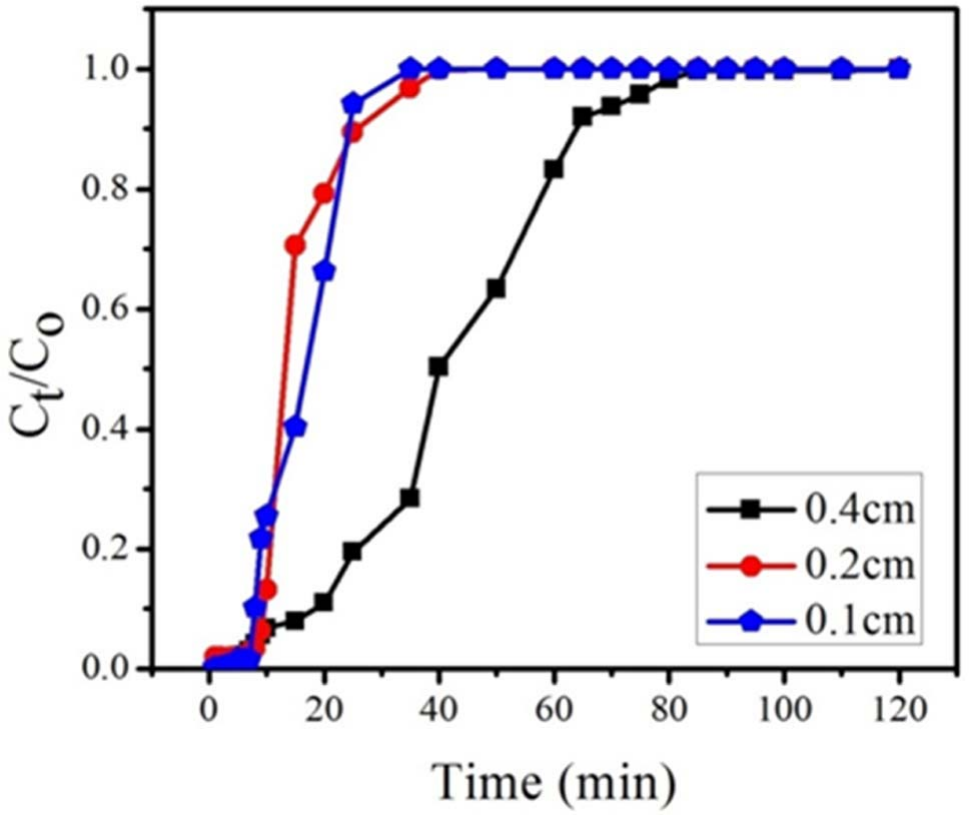
Effect of bed heights (0.4, 0.2 and 0.1 cm) on breakthrough curve at constant flow rate (1 mL min^−1^) and at constant feed concentration (300 ppm) of BPA.

The breakthrough curves for various bed heights are presented in Fig. 12 and column adsorption data is presented in Table 4. The breakthrough curve data was examined in a fixed bed system at the breakthrough point (0.1 percent influent concentration) and exhaustion point (0.9 percent influent concentration). At various bed heights such as 0.1, 0.2 and 0.4 cm (10, 20 and 40 mg), with constant flow rate of 1 mL min^−1^ and constant feed concentration of BPA (300 ppm) breakthrough curves were obtained. The results showed that with increasing bed height, the breakthrough time increased. For bed heights of 0.1, 0.2, and 0.4 cm, the breakthrough point was determined as 9, 11 and 17 min, respectively. The breakthrough time increased with increasing bed height because adsorption capacity increased with increasing the amount of GOBC; the increased adsorption capacity leads to the increased contact time, exhaustion time and influent volume. The adsorption capacity increased from 189 to 307 mg g^−1^ with increase in bed height because of the large surface area of the adsorbent and availability of more binding sites. The contact time increased in direct proportion to the height of the bed.^75^ According to these findings, a 0.4 cm bed height provided greater adsorption capacity, exhaustion duration, and treated water volume. As a result, more trials were performed with bed height of 0.4 cm.

**Table:** Fixed-bed column parameter of BPA adsorption

Bed height (cm)	Concentration (mg L^−1^)	Flow rate (mL min^−1^)	Break through time, *T*_b_ (min)	Adsorption capacity (RSD%, *n* = 3) (mg g^−1^)
0.1	300	1	9	189 ± 0.99
0.2	300	1	11	270 ± 0.86
0.4	300	1	17	307 ± 1.01
0.4	300	0.5	19	133 ± 0.63
0.4	300	0.8	18	253 ± 0.94
0.4	300	1	17	307 ± 1.01
0.4	200	1	80	481 ± 0.94
0.4	250	1	50	471 ± 1.03
0.4	300	1	17	307 ± 1.36

#### 4.2.2. Effect of flow rate on adsorption

**Fig. 13:**
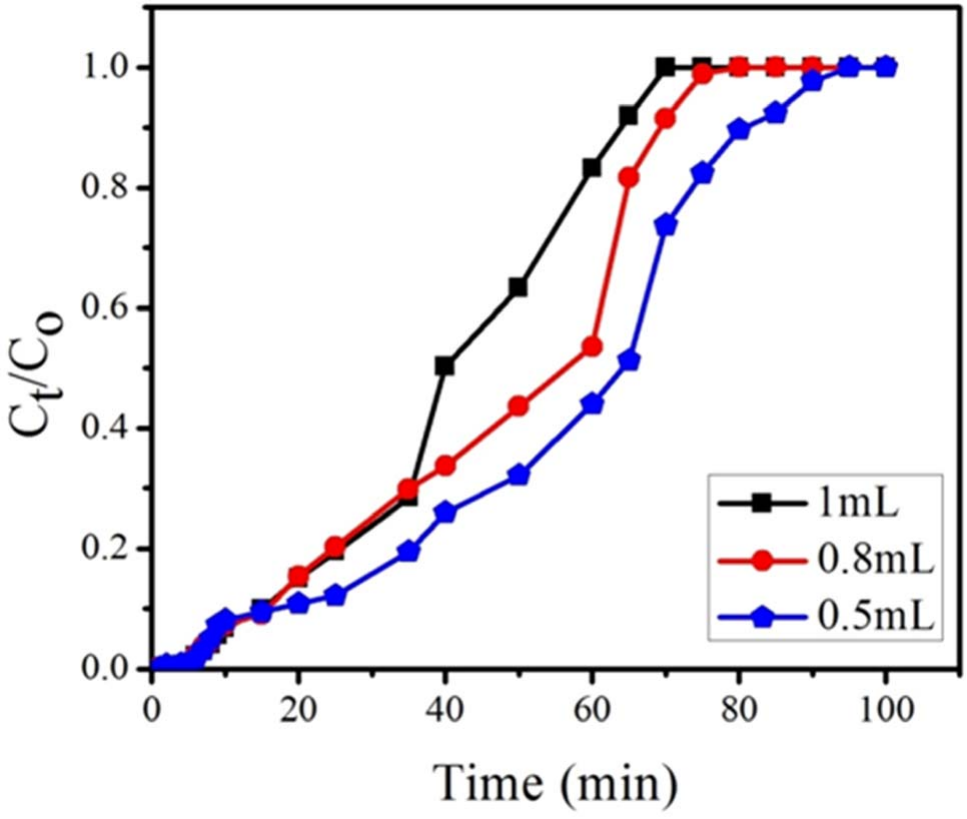
Effect of flow rates (1, 0.8 and 0.5 mL min^−1^) on breakthrough curve at constant bed height (0.4 cm) and constant feed concentration (300 ppm) of BPA.

During the adsorption of BPA using GOBC, the nature of the breakthrough curve was affected by changing the flow rate at fixed bed height. Different flow rates 0.5, 0.8 and 1 mL min^−1^ were studied through bed height of 0.4 cm and BPA solution having fixed concentration of 300 ppm. With unchanged bed height and BPA concentration, a decrease of flow rate caused an increase in breakthrough time, as shown in Fig. 13, the exhaustion time reduced from 85 to 65 minutes as the flow rate increased from 0.5 to 1 mL min^−1^. However, the adsorption capacities of BPA were found as 133, 253 and 307 mg g^−1^ with flow rate of 0.5, 0.8 and 1 mL min^−1^ as mentioned in Table 4. The adsorption capacities of GOBC column increased as the flow rate increased because of the fast adsorption kinetics of GOBC. With the increase in flow rate, the concentration of analyte decreases in the liquid phase and increases in the solid phase quickly. This phenomenon could be explained by the fact that higher flow rate allows swift interaction of analyte with the active site of adsorbent. Easier mass exchange is allowed in this process due to large fluid turbulence. Therefore, a decreased resistance of external mass transfer of adsorbent leads to increased mass transfer coefficient at higher flow rates.^76,77^

#### 4.2.3. Effect of initial concentration

**Fig. 14:**
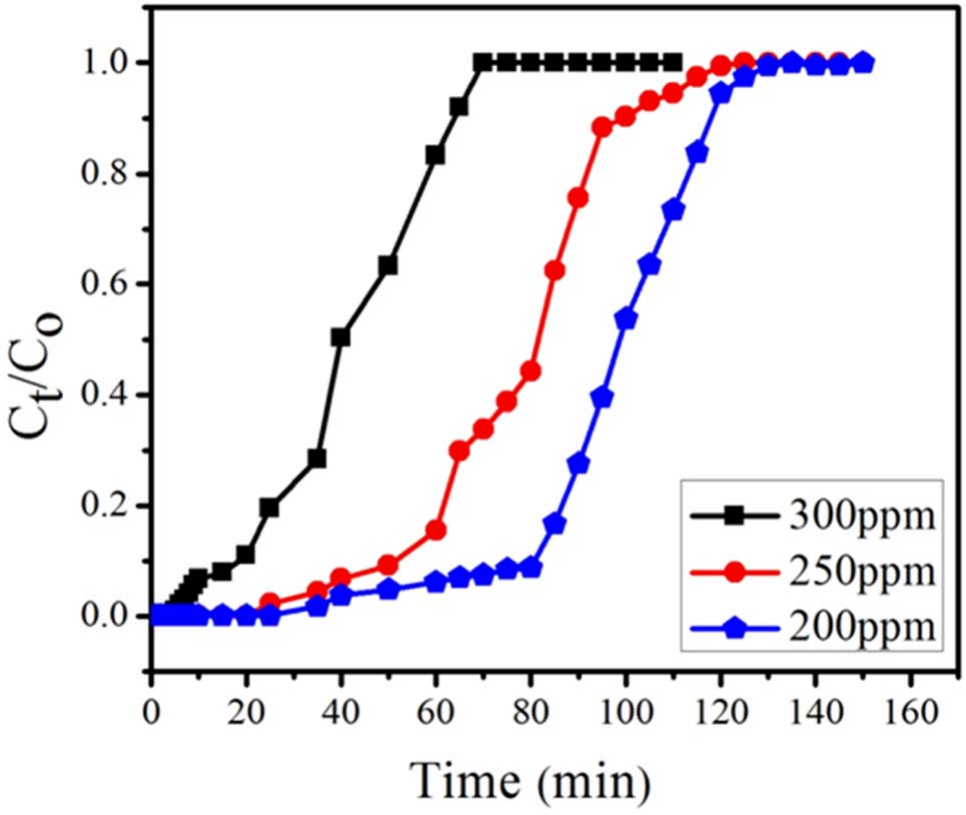
Effect of BPA concentrations (300, 250 and 200 ppm) on breakthrough curve at constant flow rate (1 mL min^−1^) and having constant bed height (0.4 cm).

The effect of initial concentration of BPA was studied at constant flow rate of 1 mL min^−1^ and bed height of 0.4 cm, using initial BPA concentrations of 200, 250 and 300 ppm (Fig. 14). Extended breakthrough curve was observed at 200 ppm with exhaustion time of 130 min. While at 300 ppm, the breakthrough curve declined with consequent fall of the exhaustion time of 45 min. These results revealed that lower concentration gradient leads to lower mass transfer coefficient of BPA while at higher concentration of BPA column saturates quickly. Similar trends were shown in other studies.^78^ Metwally *et al.*^79^ also reported similar trend using nano-sized iron(iii)–titanium(iv) mixed oxide for the removal of Co^2+^, Cd^2+^ and Ni^2+^ ions.

For evaluating the efficiency and usability of fixed-bed adsorption column for large scale processes, number of mathematical models are developed. Breakthrough curves and the performance efficiency of the GOBC fixed-bed column were studied by applying three well known mathematical models, *i.e.* Adam–Bohart model, Thomas model and Yoon–Nelson model. The detailed discussion about these mathematical models is added in ESI under Section 1S.†

## 5. Adsorption capacity of other selected organic and inorganic contaminants

Efficiency of composite material was also checked for selected organic and inorganic contaminants at optimized conditions of column such as flow rate 1 mL min^−1^, bed height 0.4 cm at ambient conditions.

### 5.1. Adsorption capacity of selected organic contaminants

The ability of GOBC fixed-bed column was also checked for the removal of other organic contaminants. Therefore, various organic contaminants such as BPS, methylene blue, methylene violate, beta-estradiol and endosulfan were also studied for their removal from aqueous environment using GOBC fixed-bed column. The GOBC fixed-bed column showed good adsorption capacities for these all contaminants as shown in Fig. 1S in ESI.† The prepared GOBC showed maximum adsorption capacity for BPA (307 mg g^−1^) and BPS (305 mg g^−1^) (Table 5). It is because these two compounds are sufficiently hydrophilic with a molecular structure comprised of two benzene rings and hydroxyl groups. The sulphonic acid functionalities in GOBC are capable of interacting with bisphenols easily. The adsorption capacity of methylene violet was found 290 mg g^−1^. The reduced adsorption capacity is observed due to bulky structure of methylene violet as compared to bisphenols. Also, the presence of negatively charged chloride ions in the structure of methylene violet and methylene blue could be responsible for the repulsion effect from GOBC as the surface of GOBC is also negatively charged. However, the adsorption capacity of methylene violet is higher than the adsorption capacity of methylene blue due to the different molecular structures. In case of endosulfan the adsorption capacity further decreased. Due to the presence of plenty of chlorine atoms in its structure; endosulfan is incapable to interact with GOBC as efficiently as bisphenols. The GOBC showed least adsorption capacity of 195 mg g^−1^ for beta-estradiol. It is due to lipophilic nature of beta-estradiol. However, it is worth mentioning that GOBC has shown good adsorption capacities for all the tested EDCs. It can serve as universal adsorbent for both hydrophilic and lipophilic organic contaminants.^80^

**Table:** Adsorption capacity of GOBC for selected organic contaminants at optimized conditions

S. no.	Name	Adsorption capacity (mg g^−1^)	RSD (*n* = 3)
1	BPA	307	±1.01
2	BPS	305	±0.421
3	Methylene violate	290	±0.320
4	Methylene blue	260	±2.012
5	Endosulfan	230	±1.56
6	Beta-estradiol	195	±3.271

### 5.2. Adsorption capacity of selected toxic metal ions

The adsorption capacity of GOBC for inorganic contaminants was also evaluated using fixed-bed column. Precisely, stock solution of 10 metals was prepared together in 1000 mL of DI water. Then stock solution was diluted to desired concentrations and column study was carried at optimized conditions. Initial and final concentrations of metals were determined by ICP-OES. The results (Fig. 2S in the ESI†) revealed that composite material shows marvelous tendency for inorganic metal ions as well. The maximum adsorption capacity of 156 mg g^−1^ was found for Pb^2+^ and the lowest for As and Cu as listed in Table 6. The GOBC showed greater tendency for Pb^2+^ ions due its greater ionic radii as compared to As and Cu. Moreover, the presence of sulphonic acid functionalities in the structure of GOBC are also capable to form complex with Pb^2+^ ions readily.^81,82^

**Table:** Adsorption capacity of GOBC for selected inorganic contaminants

S. no.	Name	Adsorption capacity (mg g^−1^)	RSD (*n* = 3)
1	Pb^2+^	156	±0.0016
2	Li^+^	136	±0.44
3	Ni^2+^	126	±0.0011
4	Co^2+^	124	±0.0001
5	Cr^6+^	118	±0.001
6	Zn^2+^	114	±0.0042
7	Cd^2+^	82	±0.0001
8	Hg^2+^	82	±1.5
9	As^5+^	72	±0.0082
10	Cu^2+^	72	±0.0002

### 5.3. Treatment of industrial wastewater

**Fig. 15:**
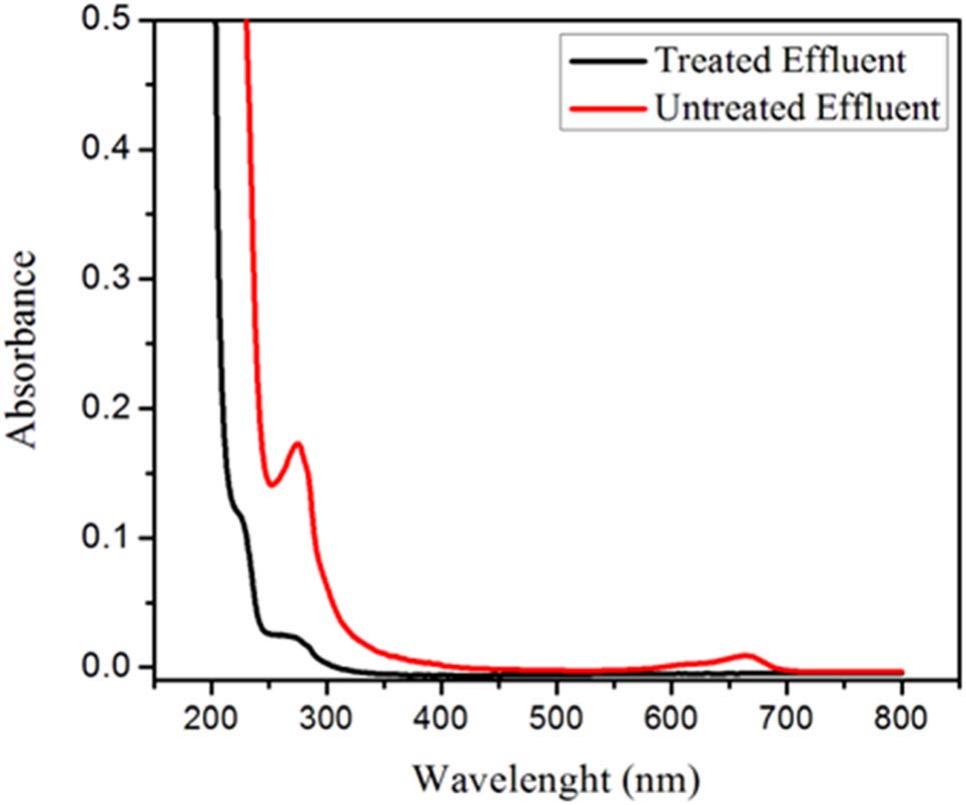
UV-VIS spectra of the untreated and treated effluent before/after column treatment through GOBC.

The efficacy of GOBC for treating polluted wastewater effluent comprising of dyes, inorganic, and organic pollutants was investigated. The wastewater samples were collected from the main drain of Kotri Industrial Area, Kotri, Sindh Pakistan. Initially, the water quality parameters were evaluated and found as pH 6.58 ± 0.32, total dissolved solids (TDS) 163 ± 3.43 mg L^−1^, total suspended solids (TSS) 555 ± 4.21 mg L^−1^, chlorides 98.5 ± 1.13 mg L^−1^, oil and grease 38 ± 2.42 mg L^−1^ and chemical oxygen demand (COD) 160 ± 4.23 mg L^−1^. The color of the industrial wastewater sample was found grey. All the water quality parameters were performed following standard methods for water quality assessment. After initial assessment of wastewater samples, the samples were spiked with known concentrations of selected organic contaminants such as bisphenol A, bisphenol S, endosulphan, beta-estradiol and dyes (methylene blue and violate). The UV-visible spectra of the effluent before and after treatment are shown in Fig. 15. When comparing the two spectra, it is obvious that the intensity of effluent spectrum after treatment was substantially decreased; proving GOBC's superior performance as fixed-bed column. The overall 87.6% removal of contaminants was achieved. These findings revealed that GOBC is an effective adsorbent for treating contaminated wastewater including a variety of contaminants. In order to further investigate the performance of GOBC, the non-spiked and spiked industrial wastewater samples were analyzed by ICP-OES and GC-MS, respectively to check the presence and removal of metal ions and organic contaminants, respectively. The details of analysis are presented in section 5.4 and 5.5.

### 5.4. ICP-OES data analysis

**Fig. 16:**
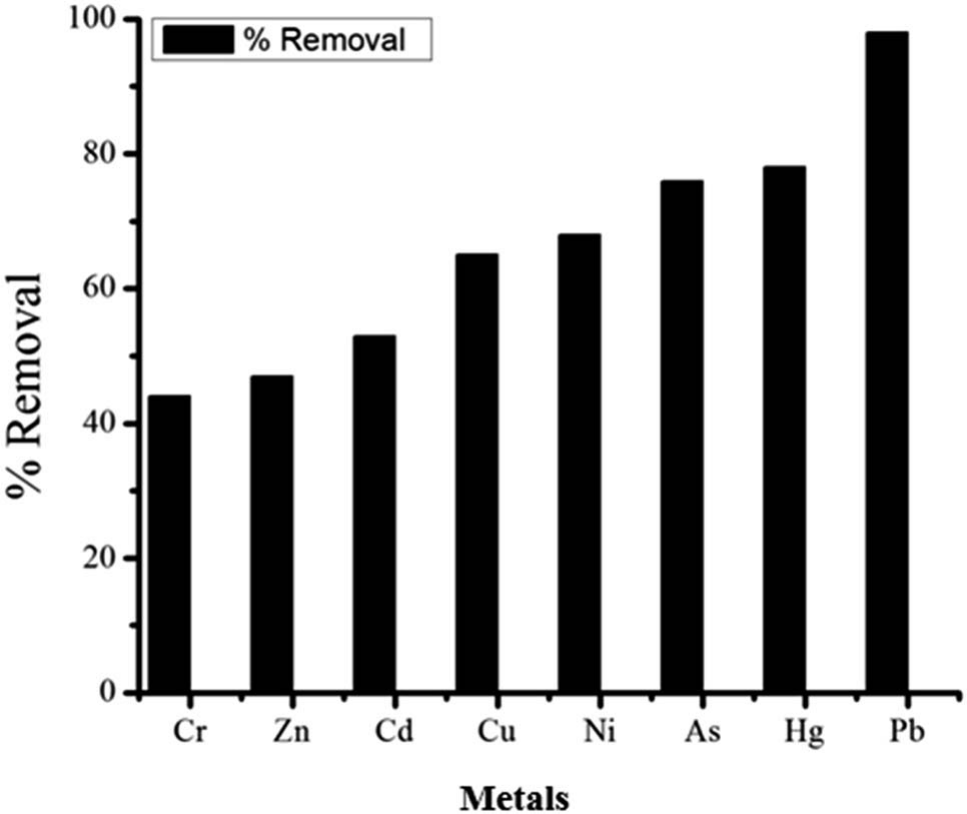
Percent removal of metals in real water samples treated through GOBC fixed column.

As shown in Fig. 16, the non-spiked industrial wastewater sample was tested before and after treatment process through the fixed-bed column having 0.4 cm bed height and constant flow rate of 1 mL min^−1^. The concentration of metals in water was determined by ICP-OES. The samples of 50 mL were used for adsorption studies. Total eight metals ions were found in the non-spiked industrial wastewater samples such as Pb^2+^, Ni^2+^, Cr^6+^, Zn^2+^, Cd^2+^, Hg^2+^, Cu^2+^, and As^5+^. The GOBC showed good capacity for the removal of these metal ions. As shown in Table 7; the maximum percent removal in non-spiked industrial wastewater samples was achieved for Pb about 96% and lowest was found for Cr 44%. In the light of these results, it can be concluded that GOBC has good affinity for Pb^2+^ due to the presence of sulphonic acid functionality in its structure. Moreover, the GOBC is capable to follow three different phenomena simultaneously that is it can offer chemical interaction due to the presence of sulphonic acid functionality; it can offer ion exchange mechanism due to the presence of Na^+^ ions and it can work as nano sieve due to the presence of plenty of nano-pores in its structure.

**Table:** Real water samples analysis for metals using ICP-OES before and after adsorption

Metals	Initial concentration (mg L^−1^)	Final concentration (mg L^−1^)	% Adsorption	RSD (*n* = 3)
Cr	0.0063	0.0035	44	±0.0317
Zn	0.0210	0.0111	47	±0.022
Cd	0.0044	0.0021	53	±0.0044
Cu	0.0005	0.0002	65	±0.0231
Ni	0.0161	0.0052	68	±0.0135
As	0.0317	0.0764	76	±0.021
Hg	0.0746	0.0164	78	±5.710
Pb	0.0027	0.0001	96	±0.005

### 5.5. GC/MS data analysis

The spiked industrial wastewater samples were also analyzed before and after adsorption by GOBC in a fixed-bed column study using GC-MS to evaluate the removal of organic contaminants. For the analysis of GC-MS the spiked industrial wastewater samples (before and after adsorption) were extracted with 3 : 1 ratio of dichloromethane and acetonitrile. The extract was injected into GC-MS installed with a HP-5MS (30 m × 0.25 mm i.d. with 0.25 μm film thickness) containing 5% phenyl methyl siloxane (model Agilent 19091S-433). The column temperature was programmed as follows; the initial temperature of column was set as 100 °C and reached to maximum temperature of 290 °C at 10 °C min^−1^. The total analysis time was 19 min. The carrier gas helium was used at pressure of 10.5 psi. The injection volume of 1 μL was used under split-less injection mode.

**Fig. 17:**
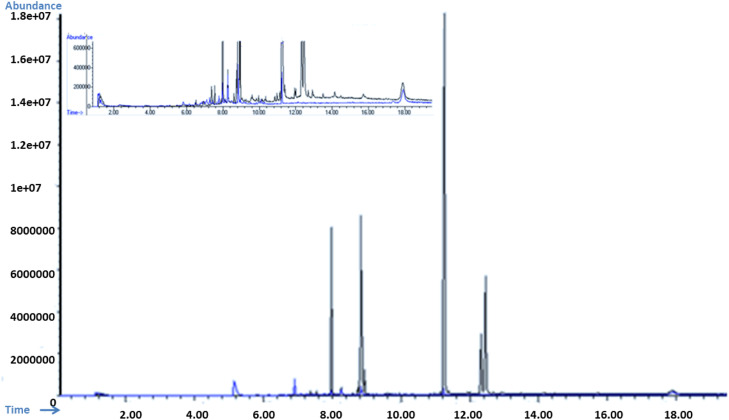
Over lay of GS-MS spectra of spiked wastewater sample before adsorption (black) and after adsorption (blue); inset: over lay spectra near to base-line.

Forty-seven types of organic compounds were detected in the industrial effluent sample. However, BPA, BPS, beta-estradiol and endosulfan were spiked in samples to check the removal efficiency in real applications. The material successfully adsorbed multiple contaminants from wastewater. The GOBC showed 90% removal efficiency for BPA and 78% for BPS, 74% for endosulfan and 92% for beta-estradiol as shown in Table 8. It is noteworthy that other organic contaminants (Table 9) were also adsorbed by the GOBC. The percent adsorption of GOBC for other organic contaminants was found as, diphenyl ether 95%, dibutyl phthalate 52%, 2,6-diisopropylnaphthalene 72%, and so on. Fig. 17 shows the overlay GC-MS spectra of industrial wastewater before and after adsorption using GOBC column. The spectra clearly shows that most of the contaminants are removed from the wastewater sample using GOBC adsorption column. Based on the present results, it can be concluded that GOBC can remove organic compounds with higher molecular weights and with complicated structures including bisphenols, pesticides, dyes, *etc.*

**Table:** GC/MS analysis of spiked industrial wastewater before and after adsorption using GOBC fixed-bed column

S. no.	Name	RT	% Adsorption	RSD (*n* = 3)
1	BPA	6.8252	90.01405	±0.394
2	Endosulfan	7.9699	74.49748	±0.071
3	BPS	8.8583	78.17097	±0.104
4	Beta estradiol	8.9456	92.32455	±0.821

**Table:** Removal of other contaminants existing in real industrial wastewater using GOBC fixed-bed column analyzed by GC/MS

S. no.	Retention time	Pollutants	Removal%
1	1.151	9-Methyl-*Z*-10-pentadecen-1-ol	11
2	1.1728	13-Octadecenal (*Z*)	39
3	1.2328	1-Pyrroline, 3-ethyl	41
4	5.1463	Diphenyl ether	98
5	6.7488	9,9-Dihydroxybicyclo[3.3.1]nonane-2,4-dione	90
6	6.9014	Disulfide, dihexyl	95
7	7.0595	2,6-Diisopropylnaphthalene	72
8	7.0977	Isopropyl myristate	41
9	7.441	1,2-Benzenedicarboxylic acid, butyl 2-ethylhexyl ester	12
10	7.7736	Hexadecanoic acid, methyl ester	13
11	7.9698	7,9-Di-*tert*-butyl-1-oxaspiro(4,5)deca-6,9-diene-2,8-dione	89
12	8.1006	Dibutyl phthalate	52
13	8.2586	10,18-Bisnorabieta-8,11,13-triene	40
14	8.771	9-Octadecenoic acid, methyl ester	56
15	8.8364	Octadecanoic acid, methyl ester	95
16	8.9454	Phenol, 4,4′-(1-methylethylidene)	75
17	9.9593	1,2-Benzenedicarboxylic acid, diisooctyl ester	15
18	11.2293	(2-Nitro-4-trifluoromethyl-phenoxy)-acetic acidhydrazide	64

### 5.6. Column re-usability

For this study, a synthetic wastewater containing EDCs such as bisphenol A, bisphenol S, endosulphan, beta-estradiol, dyes (methylene blue and violate) and toxic metals such as Pb^2+^, Li^+^, Ni^2+^, Co^2+^, Cr^6+^, Zn^2+^, Cd^2+^, Hg^2+^, Cu^2+^, and As^5+^ was used and total percent removal was analyzed using UV-Vis spectrophotometer. The composition of synthetic wastewater is given in Table 4S in ESI.† A column with a bed depth of 0.4 cm was chosen, desorption was carried out using mixture of methanol and water (25 mL) in ratio of 3 : 7 at flow rate of 1 mL min^−1^ first and then 2.5 mL of 0.1 M HCl solution was passed through the column at flow rate of 0.5 mL min^−1^. After washing with HCl column was thoroughly washed with D.I water until it was neutralized. At different time intervals concentration was monitored. After the complete removal of contaminants, the column was re-used for further adsorption process. The columns were re-used for 4 times, they showed excellent removal percentage as shown in Fig. 3S in ESI.† The obtained results indicated that the adsorbent loses efficiency of around 10% after four cycles. This loss in efficiency is insignificant when an effluent with heavy load of contaminants is passed through it. The obtained results showed the robustness of prepared material. Recently, Brião *et al.*^83^ reported 5 times reusability of clay based fixed-bed column for neodymium. However, GOBC showed good regeneration even after loading of multiple contaminants simultaneously.

### 5.7. Comparison of adsorption efficiency of GOBC with recent adsorbents

The efficiency of GOBC's adsorption was also compared with recently reported similar adsorbents (Table 10). Recently, Chatchai Rodwihok *et al.* reported ternary zinc oxide/zeolite-coal fly ash/reduced graphene oxide (ZnO/Z/rGO) nanocomposites as photocatalytic adsorbent for organic contaminants and performed adsorption experiments using methylene blue.^84^ Ferrihydrite-graphene oxide foams were also reported for the removal arsenic III from aqueous environment. The foams were able to remove 99% As(iii).^85^ Similarly, Monu Verma *et al.*^86^ also reported a one pot synthesis protocol for chitosan functionalized with EDTA and graphene oxide adsorbent. This study also targeted removal of organic and inorganic contaminants simultaneously and incorporated ethylenediaminetetraacetic acid (EDTA) for the capture of Pb^2+^ and Cd^2+^ metal ions and ciprofloxacin (CIP) and sildenafil (SDF) from wastewater.^86^ Graphene oxide-chitosan composite (GCS) based fixed bed column was also reported for the removal of 2-methylpyridine. The adsorption capacity of GCS was 7.8 mg g^−1^ for 2-methylpyridine.^87^ A poly(3-aminobenzoic acid)/graphene oxide/cobalt ferrite (P3ABA/GO/CoFe_2_O_4_) nanocomposite was reported as low-cost adsorbent by Babakir *et al.*^88^ The composite showed good adsorption capacity for Congo red dye which is 153.92 mg g^−1^. The porous materials are highly appreciated for removal of contaminants using adsorption technique. A porous composite of activated carbon and graphene oxide (AC/GO) was also reported to remove pharmaceutical compounds from the aqueous system. In this study comparison of adsorption capacity of activated carbon (AC) and AC/GO were compared. The results revealed the adsorption capacity of AC was found as 234.7 and 200.9 mg g^−1^ for BPA and paracetamol, respectively; which was slightly enhanced to 239.9 and 222.6 mg g^−1^ for BPA and paracetamol, respectively using AC/GO.^89^ It is worth mentioning that GOBC is capable of removing several organic compounds and metal ions simultaneously that reflects the outstanding capacity and selectivity of GOBC.

**Table:** Comparison of removal efficiency of GOBC with recently reported adsorbents

S. no.	Composite	Contaminants	Adsorption capacity (mg g^−1^)	Reference
1	ZnO/FA-zeolite/rGO	Methylene blue	119	84
2	FH-GO foams	Arsenic(iii)	0.46	85
3	GO-CS-EDTA	Lead(ii), cadmium(ii)	351.2, 264.1	86
4	GCS	2-Methylpyridine	7.8	87
5	P3ABA/GO/CoFe_2_O_4_	Congo red	153.92	88
6	AC/GO composite	BPA, paracetamol	239.9, 222.6	89
**7**	**GOBC**	**BPA, BPS, beta-estradiol, endosulfan, methylene blue, methylene violate and Pb, Li, Co, Cr, Zn, Cd, Hg, Cu, As**	**307, 305, 290, 260, 230, 195, 159, 136, 126, 124, 118, 114, 82, 82, 72, 72**	**Current work**

## 6. Conclusion

Graphitic materials are excellent adsorbents. The successful fabrication of graphene-based composite with tuned hydrophilicity was achieved by incorporating sulphonic acid functionality along with long chain hydrocarbon functionalities into GO. The presence of these functionalities made GOBC capable of adsorbing both hydrophobic and hydrophilic organic contaminants and toxic metal ions from real aqueous. Furthermore, the microwave method for the production of reduced graphene oxide was swift and simple. The resulting composite didn't only remove organic contaminants but also removed toxic metal ions successfully. This is because of the existence of higher number of nanopores with increased surface area of GOBC as depicted by BET results. The adsorption results of industrial wastewater showed GOBC is an excellent adsorbent that can remove both organic and inorganic contaminants simultaneously. It has excellent adsorption capacity for the maximum number of contaminants. Therefore, it can be successfully utilized in fixed-bed column or in effluent tank to treat industrial effluent.

## Conflicts of interest

All the authors declare that there is no conflict of interest about the data presented in this article.

## Supplementary Material

RA-013-D3RA02602G-s001

## References

[cit1] Programme W. W. A. , Water: A shared responsibility, Berghahn Books, 2006

[cit2] Ewis D., Ba-Abbad M. M., Benamor A., El-Naas M. H. (2022). Appl. Clay Sci..

[cit3] Guan Y.-F., Marcos-Hernández M., Lu X., Cheng W., Yu H.-Q., Elimelech M., Villagrán D. (2019). Environ. Sci. Technol..

[cit4] Su H., Wei Y., Qu X., Yu C., Li Q., Alvarez P. J., Long M. (2020). Chem. Eng. J..

[cit5] Padilla J. E., Melendez J., Barrera L. A., Wu Y., Ventura K., Veleta J. M., Islam M. T., Chavez C. A., Katla S. K., Villagrán D. (2018). J. Environ. Chem. Eng..

[cit6] Suh M.-J., Shen Y., Chan C. K., Kim J.-H. (2019). Langmuir.

[cit7] Li M., Farmen L. M., Chan C. K. (2017). Ind. Eng. Chem. Res..

[cit8] Chu C., Yang J., Huang D., Li J., Wang A., Alvarez P. J., Kim J.-H. (2019). Environ. Sci. Technol..

[cit9] Zhou R., Zhao L., Wang Y., Hameed S., Ping J., Xie L., Ying Y. (2020). TrAC, Trends Anal. Chem..

[cit10] Zhao F., Yao Y., Jiang C., Shao Y., Barceló D., Ying Y., Ping J. (2020). J. Hazard. Mater..

[cit11] Yao Y., Ping J. (2018). TrAC, Trends Anal. Chem..

[cit12] Ge J., Shi L.-A., Wang Y.-C., Zhao H.-Y., Yao H.-B., Zhu Y.-B., Zhang Y., Zhu H.-W., Wu H.-A., Yu S.-H. (2017). Nat. Nanotechnol..

[cit13] Liu Y., Huang Y., Duan X. (2019). Nature.

[cit14] Cai J., Ruffieux P., Jaafar R., Bieri M., Braun T., Blankenburg S., Muoth M., Seitsonen A. P., Saleh M., Feng X. (2010). Nature.

[cit15] Iannaccone G., Bonaccorso F., Colombo L., Fiori G. (2018). Nat. Nanotechnol..

[cit16] Mao S., Chang J., Pu H., Lu G., He Q., Zhang H., Chen J. (2017). Chem. Soc. Rev..

[cit17] Yuan W., Chen J., Shi G. (2014). Mater. Today.

[cit18] Wang L., Boutilier M. S., Kidambi P. R., Jang D., Hadjiconstantinou N. G., Karnik R. (2017). Nat. Nanotechnol..

[cit19] Prozorovska L., Kidambi P. R. (2018). Adv. Mater..

[cit20] Cai X., Luo Y., Liu B., Cheng H.-M. (2018). Chem.
Soc. Rev..

[cit21] Kim S., Ievlev A. V., Jakowski J., Vlassiouk I. V., Sang X., Brown C., Dyck O., Unocic R. R., Kalinin S. V., Belianinov A. (2018). Carbon.

[cit22] Danda G., Das P. M., Drndić M. (2018). 2D Mater..

[cit23] Wang H., Kurata K., Fukunaga T., Takamatsu H., Zhang X., Ikuta T., Takahashi K., Nishiyama T., Ago H., Takata Y. (2016). Carbon.

[cit24] Kim S., Dyck O., Ievlev A. V., Vlassiouk I. V., Kalinin S. V., Belianinov A., Jesse S., Ovchinnikova O. S. (2018). Carbon.

[cit25] Schneider G. F., Kowalczyk S. W., Calado V. E., Pandraud G., Zandbergen H. W., Vandersypen L. M., Dekker C. (2010). Nano Lett..

[cit26] Koenig S. P., Wang L., Pellegrino J., Bunch J. S. (2012). Nat. Nanotechnol..

[cit27] Surwade S. P., Smirnov S. N., Vlassiouk I. V., Unocic R. R., Veith G. M., Dai S., Mahurin S. M. (2015). Nat. Nanotechnol..

[cit28] Feng J., Liu K., Graf M., Lihter M., Bulushev R. D., Dumcenco D., Alexander D. T., Krasnozhon D., Vuletic T., Kis A. (2015). Nano Lett..

[cit29] Kuan A. T., Lu B., Xie P., Szalay T., Golovchenko J. A. (2015). Appl. Phys. Lett..

[cit30] Fan Z., Zhao Q., Li T., Yan J., Ren Y., Feng J., Wei T. (2012). Carbon.

[cit31] Yamada Y., Murota K., Fujita R., Kim J., Watanabe A., Nakamura M., Sato S., Hata K., Ercius P., Ciston J. (2014). J. Am. Chem. Soc..

[cit32] Mashiyama D., Tobe T., Ogino T. (2015). J. Phys. Chem. C.

[cit33] Nam S., Choi I., Fu C.-c., Kim K., Hong S., Choi Y., Zettl A., Lee L. P. (2014). Nano Lett..

[cit34] Ruello J. L. A. (2022). J. Environ. Chem. Eng..

[cit35] Araichimani P. (2022). Ceram. Int..

[cit36] Hijab M. (2021). Chem. Eng. Process.: Process Intensif..

[cit37] Ahmed S., Shaikh H., Solangi A., Barek J., Sirajuddin, Denizli A., Agheem M. H. (2020). Monatsh. Chem..

[cit38] Mendes C. R., Dilarri G., Bidoia E. D., Montagnolli R. N. (2020). Matéria.

[cit39] Langmuir I. (1918). J. Am. Chem. Soc..

[cit40] Freundlich H. M. F. (1906). J. Phys. Chem..

[cit41] Lagergren S. K. (1898). K. Sven. Vetenskapsakad. Handl..

[cit42] Ho Y.-S., McKay G. (1998). Chem. Eng. J..

[cit43] McKay G., Blair H., Gardner J. (1982). J. Appl. Polym. Sci..

[cit44] Dideikin A. T., Vul’ A. Y. (2019). Front. Phys..

[cit45] Soomro A. N., Shaikh H., Malik M. I., Buledi J. A., Qazi S., Solangi A. (2022). RSC Adv..

[cit46] Reynosa-Martínez A. C., Gómez-Chayres E., Villaurrutia R., López-Honorato E. (2021). Materials.

[cit47] Chen W., Yan L., Bangal P. R. (2010). J. Phys. Chem. C.

[cit48] Zahid M., Khalid T., Rehan Z. A., Javed T., Akram S., Rashid A., Mustafa S. K., Shabbir R., Mora-Poblete F., Asad M. S., Liaquat R., Hassan M. M., Amin M. A., Shakoor H. A. (2021). Membranes.

[cit49] Baheti K. G., Shah N., Shaikh S. (2012). Indian J. Pharm. Sci..

[cit50] Ren H., Yang X.-T., Yu J.-G. (2023). J. Environ. Chem. Eng..

[cit51] Mughal Z. u. N., Shaikh H., Memon S., Raza R., Shah R., Bhanger M. I. (2020). Int. J. Energy Res..

[cit52] Beydaghi H., Javanbakht M., Kowsari E. (2014). Ind. Eng. Chem. Res..

[cit53] Liu X.-Y., Huang M., Ma H.-L., Zhang Z.-Q., Gao J.-M., Zhu Y.-L., Han X.-J., Guo X.-Y. (2010). Molecules.

[cit54] Yee R. S., Zhang K., Ladewig B. P. (2013). Membranes.

[cit55] Andrijanto E., Shoelarta S., Subiyanto G., Rifki S. (2016). AIP Conf. Proc..

[cit56] Ferrari A. C., Meyer J. C., Scardaci V., Casiraghi C., Lazzeri M., Mauri F., Piscanec S., Jiang D., Novoselov K. S., Roth S. (2006). Phys. Rev. Lett..

[cit57] Ferrari A. C., Robertson J. (2000). Phys. Rev. B.

[cit58] Kudin K. N., Ozbas B., Schniepp H. C., Prud'homme R. K., Aksay I. A., Car R. (2008). Nano Lett..

[cit59] Paton K. R., Despotelis K., Kumar N., Turner P., Pollard A. (2023). Beilstein J. Nanotechnol..

[cit60] Paulchamy B., Arthi G., Lignesh B. (2015). J. Nanomed. Nanotechnol..

[cit61] Stobinski L., Lesiak B., Malolepszy A., Mazurkiewicz M., Mierzwa B., Zemek J., Jiricek P., Bieloshapka I. (2014). J. Electron Spectrosc. Relat. Phenom..

[cit62] Arnold T., Thomas R. K., Castro M. A., Clarke S. M., Messe L., Inaba A. (2002). Phys. Chem. Chem. Phys..

[cit63] Masood M. H., Haleem N., Shakeel I., Jamal Y. (2020). Res. Chem. Intermed..

[cit64] Zhao B., Liu P., Jiang Y., Pan D., Tao H., Song J., Fang T., Xu W. (2012). J. Power Sources.

[cit65] Qazi S., Shaikh H., Memon A. A., Memon S. (2020). J. Chem. Soc. Pak..

[cit66] Tkachev S., Buslaeva E. Y., Naumkin A., Kotova S., Laure I., Gubin S. (2012). Inorg. Mater..

[cit67] Hamra A., Lim H., Huang N., Gowthaman N., Nakajima H., Rahman M. M. (2020). J. Mol. Struct..

[cit68] Zhu L., Guo X., Chen Y., Chen Z., Lan Y., Hong Y., Lan W. (2022). ACS Appl. Nano Mater..

[cit69] Thommes M., Kaneko K., Neimark A. V., Olivier J. P., Rodriguez-Reinoso F., Rouquerol J., Sing K. S. W. (2015). Pure Appl. Chem..

[cit70] Bardestani R., Patience G. S., Kaliaguine S. (2019). Can. J. Chem. Eng..

[cit71] Rikhtehgaran S., Lohrasebi A. (2018). J. Nanosci. Nanotechnol..

[cit72] Kim J., Khoh W.-H., Wee B.-H., Hong J.-D. (2015). RSC Adv..

[cit73] Perrozzi F., Croce S., Treossi E., Palermo V., Santucci S., Fioravanti G., Ottaviano L. (2014). Carbon.

[cit74] Nandi B. K., Goswami A., Purkait M. K. (2009). J. Hazard. Mater..

[cit75] Lodeiro P., Herrero R., De Vicente M. S. (2006). J. Hazard. Mater..

[cit76] Goel J., Kadirvelu K., Rajagopal C., Garg V. K. (2005). J. Hazard. Mater..

[cit77] Piątkowska S., Gwadera M. (2018). Tech. Trans..

[cit78] Aksu Z., Gönen F. (2004). Process Biochem..

[cit79] Metwally S. S., Mekhamer H. S., El-Sherief E. A. (2020). Sep. Sci. Technol..

[cit80] Farooq Khan M., Ahmed H., Abdulkareem Almashhadani H., Al-Bahrani M., Ullah Khan A., Ali S., Gul N., Hassan T., Ismail A., Zahid M. (2022). Inorg. Chem. Commun..

[cit81] Lutoshkin M. A., Kuznetsov B. N., Levdanskiy V. A. (2019). Main Group Met. Chem..

[cit82] Metwally S. S., El-Sherief E. A., Mekhamer H. S. (2020). Sep. Sci. Technol..

[cit83] Brião G. d. V., da Silva M. G. C., Vieira M. G. A. (2022). Sustainable Chem. Pharm..

[cit84] Rodwihok C., Suwannakaew M., Han S. W., Lim Y. J., Park S. Y., Woo S. W., Choe J. W., Wongratanaphisan D., Kim H. S. (2023). Colloids Surf., A.

[cit85] Shanmugaraj K., Vinoth V., Pugazhenthiran N., Valdés H., Salvo C., Sepúlveda E., Mangalaraja R. V. (2023). Inorg. Chem. Commun..

[cit86] Verma M., Kumar A., Lee I., Kumar V., Park J.-H., Kim H. (2022). Environ. Pollut..

[cit87] Chatterjee R., Majumder C. (2023). J. Water Process Eng..

[cit88] Babakir B. A. M., Abd Ali L. I., Ismail H. K. (2022). Arabian J. Chem..

[cit89] Brito C. H. V., Gloria D. C. S., Santos E. B., Domingues R. A., Valente G. T., Vieira N. C. S., Gonçalves M. (2023). Chem. Eng. Res. Des..

