# Supplementary material for: Fabrication of 1-octane sulphonic acid modified nanoporous graphene with tuned hydrophilicity for decontamination of industrial wastewater from organic and inorganic contaminants
Source: RSC Adv. 2023 Jul 20;13(32):21926–44. doi: 10.1039/d3ra02602g (PMC10357182; doi:10.1039/d3ra02602g)
Supplement: RA-013-D3RA02602G-s001 [file RA-013-D3RA02602G-s001.pdf]

Electronic Supplementary Material (ESI) for RSC Advances.
This journal is © The Royal Society of Chemistry 2023

Fabrication of 1-Octane Sulphonic Acid Modified Nanoporous Graphene with tuned Hydrophilicity for Decontamination of Industrial Wastewater from Organic and Inorganic Contaminants

Shahbaz Ali Mallah[1], Huma Shaikh[1,*], Najma Memon[1], Sehrish Qazi[1]

[1]National Centre of Excellence in Analytical Chemistry, University of Sindh, Jamshoro, 76080, Pakistan

1S. BREAKTHROUGH CURVE MODELING

For evaluating the efficiency and usability of fixed-bed adsorption column for large scale processes, number of mathematical models are developed. Breakthrough curves and the performance efficiency of the GOBC fixed-bed column were studied by applying three well known mathematical models, i.e. Adam Bohart model, Thomas model and Yoon-Nelson model.

1.1S ADAM-BOHART MODEL

The surface reaction theory is connected to the Adam–Bohart model. It expresses the relationship between adsorbent, adsorption rate and residual capacity, as well as adsorbate concentration [1]. This model was chosen to account for the data from the first half of the breakthrough curve (breakpoint) [2]. It can be calculated using Equation 1.

$$ln\left(\frac{C_t}{C_o}\right) = K_{AB}C_ot - K_{AB}.N_o\left(\frac{Z}{U_o}\right) \quad (1)$$

Where k_{AB} denotes the Adam–Bohart constant (L/mg min), N_o denotes the maximum saturation concentration (mg/L) or maximal ion adsorption capacity per unit volume of adsorbent column, and Z denotes the column bed depth (cm). The volumetric flow rate over the bed cross-sectional area is used to get U_o, which is the linear flow velocity in cm/min. The plot of ln (Ct/C_o) against time yields the k_{AB} and N_o values (min). The values of N_o and K_{AB} for all breakthrough curves are shown in Table 1S. Maximum saturation concentration value N_o increases with the increase in bed depth, more binding sites become available for adsorption. The presence of a fixed-bed column in the early stages of adsorption suggested that external mass transfer dominated overall

system dynamics. For this model, the poor regression coefficient values were determined. Moreover, the Adams–Bohart model did not fit the experimental results well because, the model has the highest values for RMSE and SSE (3.89 for both) and has the lowest R^2 model value of 0.812 in comparison with the other models [3]. Therefore, it was concluded that GOBC fixed-bed column did not obey Adam-Bohart model.

Table 1S Adam-Bohart model parameters for adsorption of BPA using fixed-bed column method.

**Bed Height (cm)**	**Concentration (mg/L)**	**Flow rate (mL/min)**	**K_{AB}×10^{-4} (L/min mg)**	**N_o (mg/L)**	**R^2**
0.1	300	1	3.23	91827	0.502
0.2	300	1	2.93	53549	0.550
0.4	300	1	1.27	65872	0.804
0.4	300	0.5	1.13	31691	0.826
0.4	300	0.8	3.23	37704	0.558
0.4	300	1	1.27	65872	0.804
0.4	200	1	2.95	66806	0.905
0.4	250	1	2.32	75828	0.817
0.4	300	1	1.27	65872	0.804

SSE%<br>RMSE%<br>R^2	3.89<br>3.89<br>0.812				

1.2 THOMAS MODEL

For column studies, this is the most commonly utilized model. This adsorption model uses data from the column to determine the maximum solid-phase concentration of adsorbate on the adsorbent and the rate constant. This model is based on the Langmuir desorption and adsorption kinetics assumption that the rate driving force is a second order reversible reaction with no axial dispersion [4, 5]. Equation.2 represents the Thomas model

$$ln\left[\frac{C_o}{C_t} - 1\right] = \frac{k_{TH} q_o m}{Q} - k_{TH} C_o t \quad (2)$$

Here K_{TH} (mL/min mg) is the Thomas rate constant, q_o(mg/g) represents the maximum solid phase BPA concentration per weight of adsorbent. K_{TH} and q_o values were calculated by plotting the $\ln[C_o/Ct-1]$ against time (min). The values of K_{TH} and q_o found for all the breakthrough curves together with regression coefficient are shown in Table 2S. The q_o value rises as the bed height increases, and also the q_o value rises with increase in initial concentration of analyte and flow rate. It's because of the high adsorption driving force created by the large amount of adsorbate in the adsorbent, as well as the difference in concentration between BPA in the solution and BPA in the adsorbent. The regression coefficients for all of the breakthrough curves were found to be between 0.904 and 0.986, indicating that this model has a superior fit with the experimental data points. Thomas model fitted the experimental results very well, it has lower values for RMSE and SSE (1.82 for both) as compared to Adam-Bohart model. Also, the R^2 value of model is satisfactory (R^2 = 0.965). These results are in agreement with already reported results[6].

Table 2S Thomas model parameter for the adsorption of BPA using fixed-bed column method.

Bed Height (cm)	Concentration (mg/L)	Flow rate (mL/min)	$K_{TH}\times10^{-3}$ (mL/min mg)	q_0 (mg/g)	R^2
0.1	300	1	1.04	215	0.914
0.2	300	1	0.833	285	0.904
0.4	300	1	0.33	330	0.986
0.4	300	0.5	0.31	142	0.974
0.4	300	0.8	0.287	256	0.961
0.4	300	1	0.33	330	0.986
0.4	200	1	0.45	461	0.960
0.4	250	1	0.4	474	0.973
0.4	300	1	0.33	330	0.986
SSE%	1.82				
RMSE%	1.82				
R^2	0.965				

5.3. YOON-NELSON MODEL

It is a simple theoretical supposition that does not focus on the adsorbent's qualities, kind, or any other physical characteristics of the adsorption bed. There is a direct relationship for decreasing rate of adsorption to the adsorption and breakthrough on the adsorbent[7]. It is expressed by Equation. 3

$$ln\left(\frac{C_t}{C_o - C_t}\right) = k_{YN}.t - \tau.k_{YN} \quad (3)$$

Where K_{YN} represents Yoon-Nelson proportional constant (min^{-1}). The τ is time required for 50% adsorbate breakthrough in minutes. K_{YN} and τ value calculated through plot of $\ln(C_t/C_o\text{-}C_t)$ against time (min) (Table 3S). The 50% breakthrough time τ was found to significantly decrease with increase in initial concentration and increase in the flow rate because of fast saturation of the column. While the 50% breakthrough time value increased with increasing bed heights

because the adsorbent provided more binding sites to the adsorbate. Therefore, overall time required for exhaustion is longer with larger bed heights. The regression coefficient value is found good for this model. Yoon-Nelson model fitted the experimental results best. The fitness isbecause, the model has the lowest values for RMSE and SSE (1.52 for both) and as well have the highest R^2 model value of 0.998 incomparison with the other models. Garba et al. [6]also reported similar trends.

Thomas and Yoon-Nelson models offered a better correlation and regression coefficient, indicating that experimental data points and model theoretical assumptions are in good agreement.These results indicated that BPA adsorption is reversible and follows pseudo-second-order kinetics with no axial dispersion. Because adsorption is immediate, it does not follow the Adam-Bohart model. Table 3S shows Yoon-Nelson model parameter for the adsorption of BPA.

Table 3S Yoon-Nelson model parameter for the adsorption of BPA using fixed-bed column.

**Bed Height (cm)**	**Concentration (mg/L)**	**Flow rate (mL/min)**	**K_{YN}×10^{-3} (min)**	τ **(min)**	**R^2**
0.1	300	1	31.0	8	0.924
0.2	300	1	11.3	12	0.961
0.4	300	1	10.0	17	0.964
0.4	300	0.5	56.4	33	0.952
0.4	300	0.8	10.5	20	0.956
0.4	300	1	10.0	17	0.964
0.4	200	1	9.11	92	0.963
0.4	250	1	11.1	73	0.971
0.4	300	1	10.0	17	0.964
SSE%	1.52				
RMSE%	1.52				
R^2	0.998				

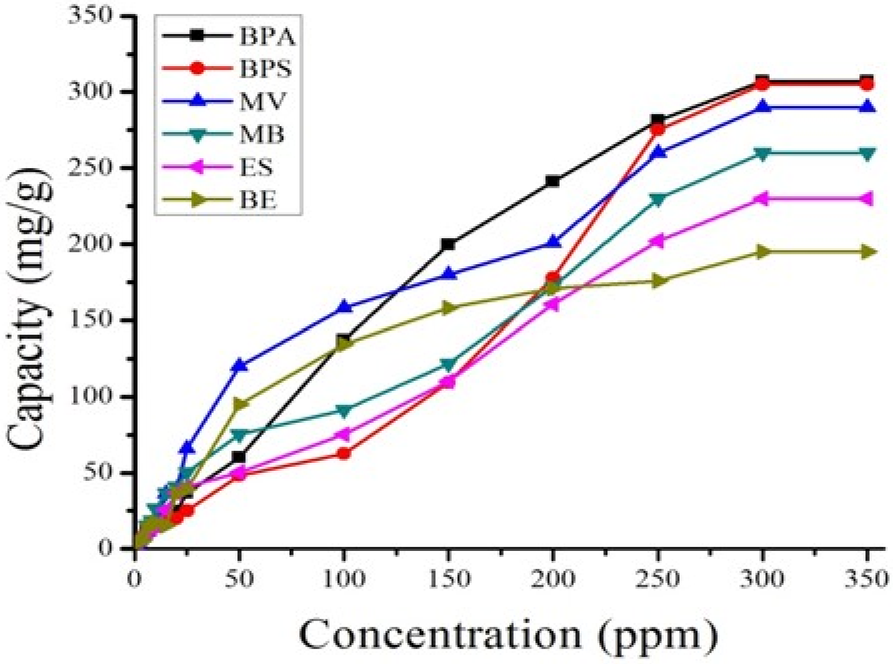

Figure 1S. Adsorption capacities of selected organic contaminants carried out at optimized column conditions

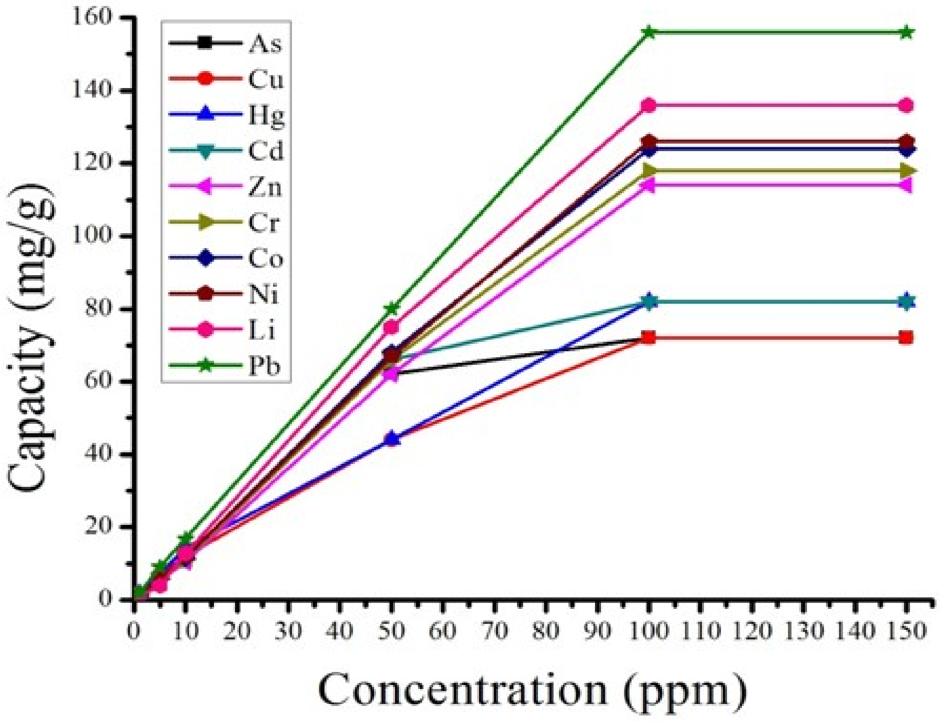

Figure 2S Adsorption capacities of selected inorganic contaminants carried out at optimized column conditions

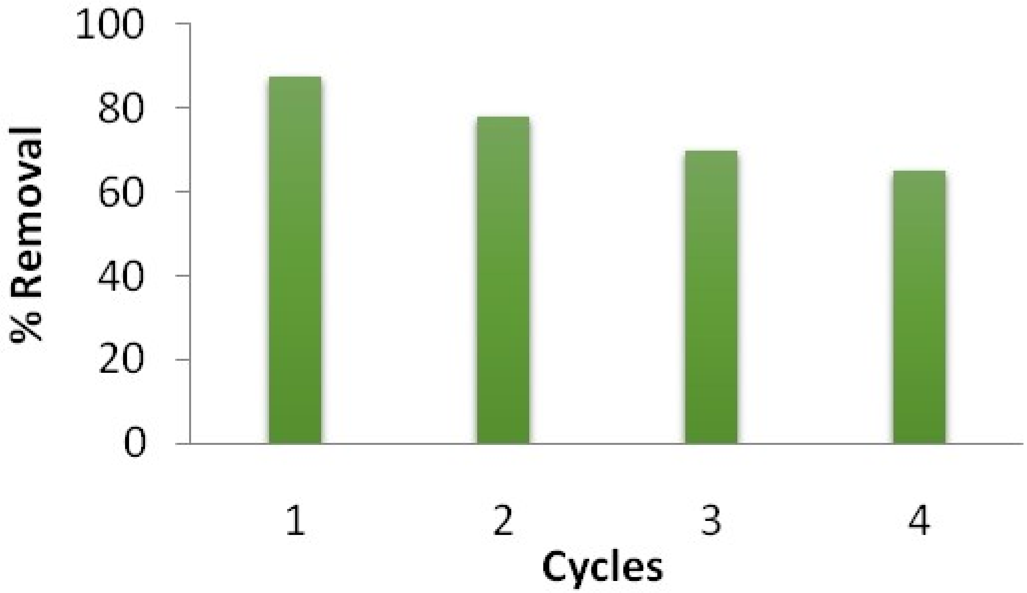

Figure 3S Re-usability study of GOBC fixed-bed column

Table 4S: Composition of Synthetic Wastewater

**Compound**	**Conc. (mg/L)**
NH_4Cl	63
NaCl	7
$CaCl_2$	4
$MgSO_4 .7H_2O$	2
K_2HPO_4	21.7
KH_2PO_4	8.5
Na_2HPO_4	25
H_3BO_3	0.57
$MnCl_2 .4H_2O$	0.25
$ZnSO_4 .7H_2O$	1.1
$FeSO_4 .7H_2O$	0.25
$CoCl_2 .6H_2O$	0.08
$Na_2MoO_4 .2H_2O$	0.015
Na_2EDTA	2.5
HEPES	1.19
pH	7.0

1. G. Bohart and E. Adams, *Journal of the American Chemical Society*, 1920, **42**, 523-544.
2. Z. Saadi, R. Saadi and R. Fazaeli, *Journal of Nanostructure in Chemistry*, 2013, **3**, 1-8.
3. A. Garba, H. Basri, N. S. Nasri, U. H. Siddiq and A. R. A. Rahman, *Malaysian Journal of Fundamental and Applied Sciences*, 2017, **13**, 803-806.
4. H. C. Thomas, *Journal of the American Chemical Society*, 1944, **66**, 1664-1666.
5. R. Apiratikul and P. Pavasant, *Bioresource technology*, 2008, **99**, 2766-2777.
6. A. Garbaa, H. Basri and N. S. Nasri, *Advanced Science Letters*, 2018, **24**, 3573-3578.
7. Y. H. Yoon and J. H. Nelson, *American Industrial Hygiene Association Journal*, 1984, **45**, 509-516.
